# A Clinical Phase 1B Study of the CD3xCD123 Bispecific Antibody APVO436 in Patients with Relapsed/Refractory Acute Myeloid Leukemia or Myelodysplastic Syndrome

**DOI:** 10.3390/cancers13164113

**Published:** 2021-08-15

**Authors:** Fatih M. Uckun, Tara L. Lin, Alice S. Mims, Prapti Patel, Cynthia Lee, Anoush Shahidzadeh, Paul J. Shami, Elizabeth Cull, Christopher R. Cogle, Justin Watts

**Affiliations:** 1 Aptevo Therapeutics, Seattle, WA 98121, USA; leec@apvo.com (C.L.); ShahidzadehA@apvo.com (A.S.); 2 Immuno-Oncology Program, Ares Pharmaceuticals, St. Paul, MN 55110, USA; 3 University of Kansas Cancer Center and Medical Pavillon, University of Kansas, Westwood, KS 66205, USA; tlin@kumc.edu; 4 Wexner Medical Center, James Cancer Hospital, The Ohio State University, Columbus, OH 43210, USA; Alice.Mims@osumc.edu; 5 Southwestern Medical Center, University of Texas, Dallas, TX 75390, USA; prapti.patel@UTSouthwestern.edu; 6 Huntsman Cancer Institute, University of Utah, Salt Lake City, UT 84112, USA; paul.shami@utah.edu; 7 Institute for Translational Oncology Research, Greenville Health System, Greenville, SC 29605, USA; Liz.Cull@prismahealth.com; 8 Division of Hematology and Oncology, Department of Medicine, College of Medicine, University of Florida, Gainesville, FL 32610, USA; Christopher.Cogle@medicine.ufl.edu; 9 Sylvester Comprehensive Cancer Center, University of Miami, Miami, FL 33136, USA; jxw401@miami.edu

**Keywords:** AML, MDS, CD123, bispecific antibody, T cells, leukemia, clinical study, APVO436

## Abstract

**Simple Summary:**

AML is a common form of blood cancer in adults. This study was undertaken to evaluate if AML patients who have failed the available standard treatment options could tolerate and potentially benefit from a new form of therapy. This new therapy activates patients’ own immune system against AML cells. The findings from this research may provide the foundation for a potentially more effective future form of standard therapy that is less likely to fail.

**Abstract:**

APVO436 is a recombinant T cell-engaging humanized bispecific antibody designed to redirect host T cell cytotoxicity in an MHC-independent manner to CD123-expressing blast cells from patients with hematologic malignancies and has exhibited single-agent anti-leukemia activity in murine xenograft models of acute myeloid leukemia (AML). In this first-in-human (FIH) multicenter phase 1B study, we sought to determine the safety and tolerability of APVO436 in R/R AML/myelodysplastic syndrome (MDS) patients and identify a clinically active recommended phase 2 dose (RP2D) level for its further clinical development. A total of 46 R/R AML/MDS patients who had failed 1–8 prior lines of therapy received APVO436 as weekly intravenous (IV) infusions at 10 different dose levels, ranging from a Minimum Anticipated Biological Effect Level (MABEL) of 0.3 mcg to 60 mcg. APVO436 exhibited a favorable safety profile with acceptable tolerability and manageable drug-related adverse events (AEs), and its maximum tolerated dose (MTD) was not reached at a weekly dose of 60 mcg. The most common APVO436-related AEs were infusion-related reactions (IRR) occurring in 13 (28.3%) patients and cytokine release syndrome (CRS) occurring in 10 (21.7%). The single dose RP2D level was identified as 0.2 mcg/kg. Preliminary efficacy signals were observed in both AML and MDS patients: Prolonged stable disease (SD), partial remissions (PR), and complete remissions (CR) were observed in R/R AML patients as best overall responses to APVO436 at the RP2D level. Three of six evaluable MDS patients had marrow CRs. The safety and preliminary evidence of efficacy of APVO436 in R/R AML and MDS patients warrant further investigation of its clinical impact potential.

## 1. Introduction

AML is the most common form of adult acute leukemia, with >20,000 estimated new cases and >11,000 deaths in the United States (US) for 2021 (SEER Program, www.seer.cancer.gov; last accessed on 13 August 2021). The rate of new cases of AML is 4.3 per 100,000 men and women per year, and the death rate is 2.8 per 100,000 men and women per year. Several targeted medicines, such as FLT3 inhibitors, IDH1/2 inhibitors, and BCL-2 inhibitor Venetoclax, as well as biotherapeutics (e.g., antibody-drug conjugates) for AML therapy have been developed and approved by the FDA over the last 10 years [1,2,3,4,5,6,7,8,9,10,11,12,13,14,15,16]. Despite these recent advances in therapy, more than 50% of patients who achieve a remission eventually relapse, and the five-year overall survival remains at 29.5%. Furthermore, the prognosis is very poor in patients who relapse after frontline induction therapy, with <10% surviving five years after the recurrence of their leukemia [8,9,10,11,12,13,14,15,16]. There is an urgent and unmet need for effective new treatment modalities for relapsed AML [8,9,10,11,12,13,14,15,16].

The α-chain of IL-3 receptor, also known as the CD123 antigen, is broadly expressed on a majority of leukemic blast cells [17,18,19,20]. CD123 is also expressed on clonogenic human AML blast cells, including the leukemia-initiating candidate leukemic stem cell populations capable of causing systemic leukemia in xenografted immunodeficient mice [17,18,19]. CD123 expression in AML is associated with both higher cell division activity and anti-apoptotic resistance at a cellular level and with a poor prognosis in clinical settings [17,21,22]. Several biotherapeutic agents targeting CD123 have been clinically evaluated against AML, including the CD123-directed recombinant human IL3 fusion toxin Tagraxofusp (SL-401); bispecific antibodies targeting CD123 antigen, such as bispecific T-cell engagers; dual affinity retargeting antibodies; bispecific killer cell engagers; and trispecific killer cell engagers [6,23,24,25,26,27,28,29,30].

**Figure 1:**
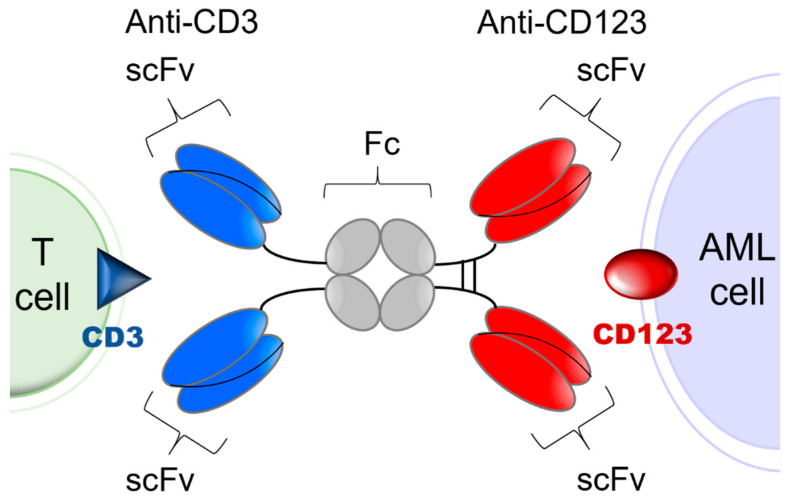
Simplified schematic explaining the mode of action of APVO436. APVO436 targeting CD123 on AML cells and redirecting CD3^+^ T cells to the close vicinity of the target leukemia cells. APVO436 is a humanized bispecific antibody that targets both CD123 and CD3. It is composed of two sets of binding domains linked to a human IgG1 Fc domain. The CD123 binding domain is a fully human scFv directed against human CD123. The CD3 binding domain is a humanized scFv that binds human CD3. The Fc region has been engineered to minimize complement fixation and interaction with Fcγ receptors.

CD3-engaging bispecific antibodies bring cytotoxic T cells (CTLs) to the close vicinity of target tumor antigen carrying AML cells to create “cytolytic synapses” as a short bridge between AML cells and CTLs, which triggers their activation and destruction of targeted AML cells. Because their activity requires concomitant binding to both the target antigen on leukemia cells and the CD3 antigen on T cells, the risk of toxicity due to broad T cell activation as well as anergy are mitigated [27,28,29]. APVO436 is a recombinant T cell engaging humanized bispecific antibody designed to redirect host T cell cytotoxicity in an MHC-independent manner to CD123-expressing blast cells from patients with hematologic malignancies [30,31,32,33] (Figure 1). APVO436 induced concentration-dependent lysis of CD123⁺ human AML cells in the presence of human T cells and triggered rapid T cell activation and proliferation with limited cytokine release [30,31,32,33]. In the presence of human T cells that were co-administered, APVO436 exhibited potent and dose-dependent single-agent anti-leukemic activity, improving the survival outcome in a NOD/SCID mouse xenograft model of human AML at dose levels ≥0.02 μg/mouse (=1 mcg/kg; human equivalent dose (HED) = 0.08 mcg/kg) [34]. Maximal anti-leukemic activity was observed at an HED of 0.4 mcg/kg (viz. 0.1 mcg per 20-gram mouse = 5 mcg/kg) [34]. The size and Fc domain of APVO436 contribute to a favorable in vivo pharmacokinetics (PK) profile with an elimination half-life of >7 days in mice and >3 days in cynomolgus monkeys [30,31,32,33]. APVO436 recognizes both CD3 and CD123 antigens in cynomolgus monkeys. Single IV injections of APVO436 ranging from 0.25 mg/kg to 1 mg/kg were well tolerated in cynomolgus monkeys [30,31,32,33]. A repeat-dose IV administration of APVO436 to cynomolgus monkeys for 4 weeks was also well tolerated at levels of 0.5, 2.5, and 10 mg/kg/dose with no clinical or laboratory evidence suggestive of systemic toxicity and no histopathologic evidence suggestive of treatment-emergent organ damage [30]. The derived no-observed-adverse-effect level (NOAEL) was 10 mg/kg with a mean C_max_ of ≈200 mcg/mL and a mean systemic exposure level (AUC) of ≈15,000 mcg/h/mL [30]. The primary purpose of the present multicenter phase 1B study was to evaluate the safety and tolerability of APVO436 in R/R AML patients and identify a clinically active recommended phase 2 (RP2D) level for its further clinical development as a new biotherapeutic agent against AML. We also evaluated the tolerability and single agent activity of APVO436 in the limited number of R/R MDS patients who were enrolled in the study.

## 2. Materials and Methods

Investigational Medicinal Product. APVO436 is a humanized bispecific antibody with an estimated molecular weight (MW) of 161 kDa that targets CD123 and CD3ε [23,30,31]. It is a glycosylated homodimeric antibody composed of two sets of binding domains linked to a human immunoglobulin (Ig) G1 fragment crystallizable (Fc) domain (Figure 1). The two chains of the homodimer are linked through inter-chain and intra-chain disulfide bridges. The CD123 binding domain is a fully human single chain variable fragment (scFv) directed against human CD123. The CD3 binding domain is a humanized scFv derived from a murine antibody that binds human CD3. The Fc domain serves to extend the half-life of APVO436 by preventing first-pass clearance through the kidney. The Fc region has been engineered to minimize complement fixation and interaction with Fcγ receptors. For this study, the cGMP lot of the APVO436 drug substance (DS) (Lot 250A17-01) was manufactured under cGMP conditions by the contract manufacturer KBI Biopharma, Inc (Durham, NC, USA 27704), in a single 2000 L bioreactor using a CHO-K1/SV cell line master cell bank (MCB) transfected with the pEE12.4 expression plasmid (Lonza Biologics GS System^TM^) encoding APVO436. The MCB was produced at BioReliance (Frederick, MD) and stored at BioReliance and at Charles River Laboratories (Malvern, PA) in liquid nitrogen (vapor phase).

The bispecific antibody was purified from the culture supernatants using multiple rounds of size exclusion and affinity chromatography. The final formulation of APVO436 was prepared by adding polysorbate 80 and sucrose to the diafiltered-retentate intermediate. The resulting final DS formulation contains 5 mM sodium succinate, 6.5% sucrose (w/v), and 0.02% (w/v) polysorbate 80. The targeted final concentration of APVO436 in DS was 2.0 mg/mL. All raw materials used in the production of the DS were purchased from approved vendors and meet USP/multi-compendial requirements where applicable. No serum or other animal products or by-products are used in manufacture of APVO436 DS. The final drug product was prepared at Ajinomoto Althea 11040 (San Diego, CA) using pre-formulated APVO436 DS shipped from KBI Biopharma under temperature-controlled conditions (−75 ± 10 °C).

Study Design and Eligibility Criteria. The clinical trial of APVO436 (Title: “Phase 1B Open-label, Dose Escalation and Dose Expansion Study of APVO436 in Patients with Relapsed or refractory (R/R) AML or High Grade MDS”) was designed as a multiple-dose phase 1B dose escalation study in patients with relapsed AML and high-risk MDS. It was registered in the clinical trial database ClinicalTrials.gov (accessed on 17 July 2018) with the identifier number NCT03647800. Patients in cohorts 1–10 received APVO436 IV for six 28-day cycles. Patients were permitted to stay on study drug for up to a total of 6 cycles until disease progression, unacceptable toxicities, or withdrawal of consent. At the discretion of the treating physicians and if no contraindications existed, patients were allowed to receive up to 36 cycles of AVPO436 if the physician determined that the patient was tolerating and benefiting from the treatments.

Patients and Patient Disposition. A total of 58 R/R adult AML/MDS patients were screened; 12 patients were screen failures, and the remaining 46 eligible patients were enrolled in the study. Eligibility criteria are detailed in the Supplementary Materials. Eligibility required adequate performance status with an Eastern Cooperative Oncology Group (ECOG) performance score of ≤2. Patients with acute promyelocytic leukemia (APL) with t(15;17), CNS leukemia, other active systemic malignancies, graft versus host disease (GvHD) or autoimmune disorders requiring immunosuppressive therapy, or uncontrolled active infections were excluded. Patients were required to not have any residual unresolved Grade >1 AEs according to National Cancer Institute Common Terminology Criteria for Adverse Events (NCI CTCAE) that resulted from previous standard or experimental treatments and were clinically significant. White blood cell (WBC) count had to be ≤25,000 cells/mm^3^, and patients were allowed to receive hydroxyurea to bring their WBC count down prior to and during the first cycle of treatment with study drug if necessary.

Study Conduct. The study was performed under IND 135552 at 10 centers in the US as an open-label study sponsored by Aptevo Therapeutics (see Supplementary Materials: Supplemental Methods). The study execution, including clinical monitoring, medical monitoring, pharmacovigilance, data management, and biostatistics, was supported by a Clinical Research Organization (CRO) according to a Transfer of Obligations Agreement.

The starting dose in Cohort 1 was 0.3 mcg (≈0.005 mcg/kg for a 60 kg patient), which was the Minimum Anticipated Biological Effect Level (MABEL) based on T cell activation assays [35]. The assigned weekly target dose levels for cohorts 2–10 were 1 mcg, 3 mcg, 9 mcg, 18 mcg (cohort 6A), 12 mcg (cohort 6B), 24 mcg, 36 mcg, 48 mcg, and 60 mcg, respectively. A 3+3 design was used to guide the dose escalation. In each cohort, eligible AML/MDS patients were assigned to receive a designated flat dose of APVO436 as a single agent via weekly intravenous infusions. APVO436 was administered according to an intra-patient step-up strategy to reduce the risk for cytokine release syndrome (CRS) that was implemented in cohort 5 with a protocol amendment. Table S1 lists all 46 patients who received APVO436 by dose cohorts. All patients in a specific dose cohort were required to complete one cycle of therapy and an evaluation for AEs and dose-limiting toxicities (DLTs) during a 28-day DLT observation period, as well as the safety data reviewed with the Investigators and the Safety Review Committee (SRC) before enrollment in the next dose cohort could begin. Dose escalation was performed according to Table S2. For each patient in cohorts 1 through 4, APVO436 was infused over approximately 20 to 24 h for the first dose (C1D1), over 8 h for the second dose (C1D8), over 6 h for the third dose (C1D15), and over 4 h for all subsequent doses (C1D22 and onwards). Stepped dosing was introduced, starting in cohort 5, to mitigate against the development of infusion-related reactions (IRR) and cytokine release syndrome (CRS). After the first cycle, most patients received their treatments as an outpatient.

AEs were graded according to National Cancer Institute (NCI) Common Terminology Criteria for Adverse Events Version 5.0 (NCI CTCAE v5.0). CRS was graded and managed, as detailed in Tables S1 and S2, respectively [36]. Response criteria of the International Working Group (IWG) were used for assessment of MDS patients. Standard European LeukemiaNet (ELN) 2017 criteria were used for response assessments in AML patients [37]. Clinical safety laboratory tests were performed according to standard methods. DNA sequencing for molecular profiling of leukemic blast cells was performed using the Genoptix (Carlsbad, CA) platform.

Ethics Statement and Study Approval. The study protocol was approved by the WCG-Central Institutional Review Board (IRB) (OHRP/FDA registration number: IRB00000533) and the local IRB at participating centers (see Supplemental Materials for the complete list of the sites where patients were treated). The Central IRB-approved study/protocol number was 20181730. The study was performed in compliance with the International Conference on Harmonization (ICH) guidelines for Good Clinical Practice (ICHE6/GCP). Each patient provided written informed consent (ICF) prior to enrollment.

Statistical Analyses. Standard statistical methods were applied for the analysis of the clinical data. Survival data were analyzed by the Kaplan–Meier method using the GraphPad Prism 9 statistical program (GraphPad Software, LLC, San Diego, CA). Log-rank statistics was used to compare the differences between patient subgroups [38,39,40]. 

## 3. Results

Patient Characteristics. Forty-six patients with R/R AML or MDS were enrolled in the study between 15/05/18 and 04/06/21. The date of data cutoff was 22 July 2021. The baseline patient characteristics are shown in Table 1. Thirty-nine patients (84.8%) had R/R AML, and seven had R/R MDS. The median age was 69 years (mean ± SE: 65.4 ± 2.0 years; range: 18–82 years). Patients had failed 1–8 prior lines of therapy (mean ± SE: 3.2 ± 0.3). A total of 9 patients (19.6%) had relapsed/progressed after 1 prior line of AML/MDS-directed therapy, 14 patients (30.4%) after 2 lines, 6 patients (13.0%) after 3 lines, and 16 patients (34.8%) after 4 or more prior lines of therapy (Table 1). Patients received 1–43 weekly doses of APVO436 (median: 7 doses; mean ± SE: 11 ± 2 doses) (Table 1). The cohort-specific APVO436 dose assignments and exposure data are detailed in Tables S1 and S2, respectively. A total of 33 of 46 patients (71.7%) died or were transferred to hospice care, and the median overall survival (OS) was 178 days, consistent with the generally poor prognosis of R/R AML and MDS patients (Figure S1).

Safety. APVO436 exhibited a favorable safety profile with acceptable tolerability and manageable side effects (see also Supplementary Materials, including Tables S1–S11). The MTD was not reached at a weekly flat dose of 60 mcg (Cohort 10), which was tolerated by all four patients enrolled without any DLTs or grade 3–4 AEs. The cumulative cycle dose levels in cohort 10 were 96 mcg for cycle 1 and 240 mcg for subsequent cycles. The single dose RP2D level was identified as 18 mcg flat dose (cohort 6; ≈0.2 mcg/kg based on the body weights of the patients enrolled) (Table S2), which was 30% of the cohort 10 dose level. This dose corresponded to the actual mean dose administered to six patients in cohort 6A and 3 patients in cohort 6B, as well as to the combined cohort A+B patients (N = 9) (Table S2). The cumulative cycle dose at this RP2D level will be 54 mcg for cycle 1 with step-dosing/weekly ramp-up (first dose: 6 mcg; second dose: 12 mcg; third dose: 18 mcg; fourth dose: 18 mcg) and 72 mcg (18 mcg weekly dose × 4) for subsequent cycles, which were substantially lower than the cycle dose levels in cohort 10 that were tolerated without DLTs. All treatment-emergent AEs (Table S3), including grade ≥3 AEs (Table S5), are detailed in the Supplementary Materials.

The incidence of each APVO436-related CTCAE grade ≥3 AEs is shown in Table 2. The most common grade ≥3 AEs suspected to be APVO436-related were grade 3–4 CRS occurring in 4 of 46 patients (8.7%), grade 3–4 anemia occurring in 2 of 46 patients (4.3%), and IRR occurring in 2 of 26 patients (4.3%). The details of all encountered APVO436-related grade ≥3 AEs are shown in Table S6. A single grade 5 AE was encountered in a patient who developed grade 2 CRS and subsequently a fatal (grade 5) acute renal failure.

No hematologic DLT was observed in any of the 10 dose cohorts. Ten patients experienced 12 episodes of grade 3 febrile neutropenia, and each one of these 12 episodes was reported as not related to APVO436 (Table S7). One patient (2.2%) who was treated in cohort 4 was reported to have a decreased platelet count (grade 4) related to APVO436 with no other reports of APVO436-related thrombocytopenia in any of the remaining 45 patients (Table 2 and Table S4).

APVO436-related SAEs were encountered in 13 of the 46 patients (28.3%) and were most commonly related to CRS (7 of 13 SAE cases, affecting 15.2% of the safety population) and IRR (3 of 17 cases, affecting 6.5% of the safety population) (Table 3). The remaining three cases included a case of possibly related sepsis that was resolved within 5 days, generalized weakness that was resolved after 28 days, and grade 1 neurotoxicity that was resolved within 2 days. One patient in cohort 4 developed a fatal acute kidney failure on day 55, 12 days after the first dose in cycle 2, which was complicated by a grade 2 CRS (Table 3). The incidence of each APVO436-related SAE is shown in Table S8. These SAE did not show any dose dependence (Table S9). The average (mean ± SE) dose levels were 0.23 ± 0.19 mcg/kg for patients who experienced SAE (N = 13) and 0.31 ± 0.05 mcg/kg for patients who did not experience any SAE (N = 33; *p* = 0.4) (Table S9). Gender, age, race, or diagnosis did not predict SAE (Table S9). The incidence of SAE was 29.2% (7 of 24 patients) for male patients and 27.3% (6 of 22 patients) for female patients (Χ^2^ *p*-value: 0.9). A total of 11 of 39 (28.2%) Caucasians and 2 of 7 (28.6%) non-Caucasians developed SAE (Χ^2^ *p*-value 1.0). None of the seven MDS patients and 13 of the 39 (33.3%) AML patients developed SAE; all 13 patients who experienced a APVO436-related SAE had R/R AML. However, the very small number of MDS patients did not allow for a reliable statistical analysis (Fisher’s exact test statistic value: 0.2; not significant).

APVO436-related transient neurotoxicity occurred only in 5 of 46 patients (10.9%). It occurred during the first cycle in four of the five patients and in cycle 8 in the remaining patient. It was mild with grade 1 AEs including headache, tremor, dizziness, lethargy, insomnia, memory loss, and confusion. A single case of grade 3 confusion (unique patient number (UPN)31 in cohort 7) was encountered on the first day of treatment and resolved within a day. In an abundance of caution, APVO436 was permanently discontinued in two of the cases (Table S10). Neurotoxicity did not show any dose dependence (Table S11). Gender, race, age, absolute lymphocyte count, or percentage of lymphocytes in peripheral blood did not predict neurotoxicity (Table S11). Neurotoxicity occurred in three patients who also experienced CRS (UPN12, UPN20, UPN31) and in two patients (UPN28, UPN46) who did not develop CRS (Table S10). Conversely, of 10 patients who developed CRS, 7 did not experience any neurotoxicity.

CRS was observed in 10 of 46 patients (21.7%) treated with APVO436 (Table S4). It occurred in cycle 1 in four patients, in cycle 2 in two patients, in cycle 3 in one patient, in cycle 5 in one patient, and in cycle 6 in two patients. It was reported as an SAE in 7 (70%) of the patients (Table 3, Tables S4 and S8). A total of 3 of 10 patients had grade 3 CRS and one patient had grade 4 CRS. Five patients developed grade 1–2 CRS that was resolved within 1–4 days. One patient with grade 2 CRS subsequently developed acute kidney failure (grade 5) with fatal outcome.

### Efficacy

**Figure 2:**
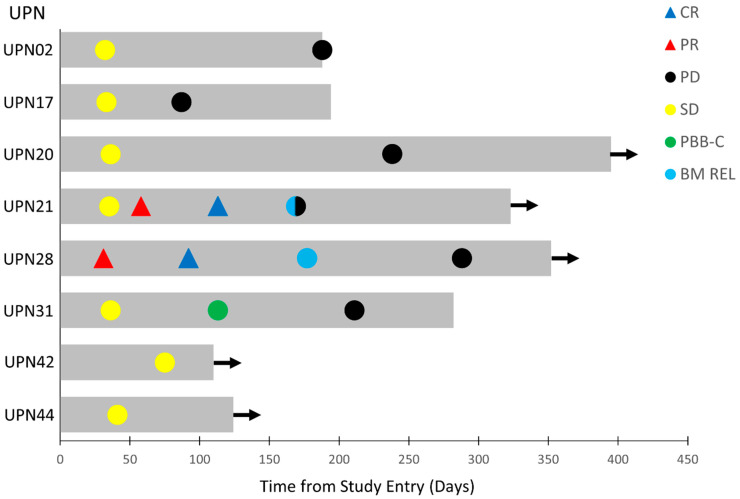
Swimmer plot of best overall responses of the eight-patient favorable response population of R/R AML patients. The onset and duration of SD, PR, CR, clearance of peripheral blasts (PBB-C), bone marrow relapse (BM REL), and onset of PD are indicated with specific symbols. Arrow: alive. See Table 4 and text for additional details.

The clinical anti-leukemia activity of APVO436 in evaluable R/R AML patients was also assessed within the confines of a phase 1 setting. Of the 39 R/R AML patients, 34 were evaluable for surrogate response measurements. Twelve patients (35.3%) had progressive disease (PD) and died of leukemia between 29 and 70 days (median: 43 days). A total of 22 of these 34 patients (64.7%) had stable disease (SD) as their best overall response. In 8 patients of these 22 patients, corresponding to 23.5% of the evaluable 34 AML patients, SD was achieved between 31 and 75 days after study entry and lasted >3 months (Table 4). Seven of eight had failed 2–4 prior lines of anti-AML therapy, and one 76-year-old patient had relapsed after achieving a remission on frontline Venetoclax plus decitabine therapy. They were enrolled 7–39 days after documentation of progressive leukemia or leukemic relapse. The onset and duration of the SD, PR, or CR in these eight patients is illustrated by the Swimmer plot depicted in Figure 2. Time-to-progression ranged from 87 to 238 days (median: 177 days) (Table 4). Of these eight patients with a favorable response to APVO436, one (UPN31) had clearance of peripheral blasts with >50% decrease in the bone marrow (BM) blast percentage. Notably, two primary AML patients with >25% BM blasts and unfavorable cytogenetics (del 5q and monosomy 7 in UPN28) and/or adverse risk group genomic mutations (TP53 mutation in UPN28 and ZRSR2 in UPN21) achieved a PR at 58 days and 75 days, respectively, which deepened to a CR with full hematologic recovery at 92 and 113 days, respectively (Figure 2).

UPN28 had developed PD after Venetoclax + Decitabine therapy, while UPN21 had failed three lines of prior anti-AML therapy (Table 4). On day 1 of cycle 5 (C5D1), UPN28 achieved CR with full hematologic recovery, including an ANC of 2.8 × 10^9^/L, platelet count of 141,000/µL, hemoglobin of 9.8 g/dL with 0% blasts in the BM, and no circulating blasts. He relapsed 85 days later with 10% BM blasts on day 1 of cycle 7 (C7D1), no circulating blasts, a WBC of 4.2 × 10^9^/L, ANC of 3.0 × 10^9^/L, platelet count of 139,000/µL, and Hgb of 10.0 g/dL (Figure 2). He continued to have SD until cycle 10, when he started showing a gradual increase in BM blast percentage with a concomitant drop in ANC and platelet count. UPN21, who had 33% BM blasts at screening and 46% BM blasts on day 1 of cycle 2, was in PR with 8% BM blasts on day 1 of cycle 3 along with an increase of ANC to 2.0 × 10^9^/L, a platelet count of 106,000/µL, and Hgb of 11.2 g/dL. On day 1 of cycle 5, he was in CR with 4% blasts, no circulating blasts, ANC of 2.8 × 10^9^/L, platelet count of 134,000/µL, and Hgb of 12.2 g/dL. He relapsed 57 days later with 66% BM blasts or a noticeable drop of peripheral counts, including an ANC of 1.6 × 10^9^ L, platelet count of 113,000/µL, and Hgb of 11.7 g/dL (Figure 2). One patient (UPN31) had a complete clearance of peripheral blasts at 113 days (from 21% pre-treatment to 14% in cycle 2, 2% in cycle 3, and 0% in cycle 4) and a >50% decrease of pretreatment BM blast percentage (from 78% prior to treatment to 37% post-treatment) followed by a sustained SD (Figure 2, Table 4).

As shown in Table S12, there was a trend towards a higher age for patients who had a favorable response. The median age among the eight patients with favorable responses was 74.5 years, whereas the median age among non-responders was 65.0 years (*p* = 0.079, Table S12). Further, we observed a trend towards a greater proportion of patients among responders being male, whereas more non-responders were female (Table S12, *p* = 0.095). Patients who had a favorable response were more likely to continue APVO436 therapy and received a significantly higher cumulative dose than non-responders (*p* < 0.0001). Patients with a favorable response had a significantly longer time to progression (*p* < 0.0001).

**Figure 3:**
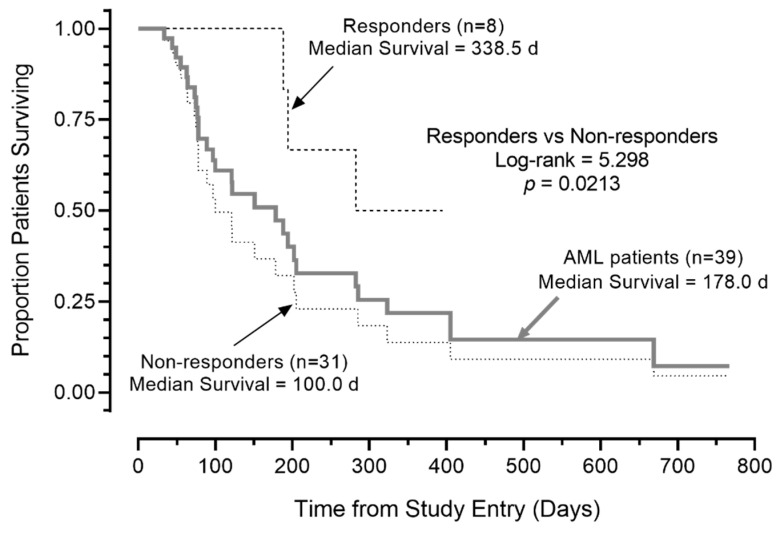
Survival outcome of AML patients according to response to APVO436. Depicted are the overall survival curves of the 8 patients’ favorable responses, 31 patients who did not respond, and all 39 patients combined. Favorable responses of CR, PR, or SD ≥ 3 months was associated with improved overall survival in R/R AML patients treated with APVO436 monotherapy.

Notably, the median OS was >300 days for the eight R/R AML patients with a favorable response (prolonged SD and PRs/CRs). Five of the eight patients remained alive at 110, 124, 323, 352, and 395 days (Table 4, Figure 2). By contrast, the median OS for the remaining 31 AML patients in the intent to treat patient population (including five who were not evaluable for response) was 100 days (95% CI: 49.8–150.2), and 24 of 31 (77.4%) died. This difference in survival outcome of favorable responders vs. non-responders was statistically significant (log-rank χ^2^ = 5.298, *p* = 0.021; Figure 3). Likewise, the survival outcome of the favorable responders was significantly better than the OS of 26 non-responders who were evaluable for response determinations, whose median OS was 121.0 days (95% CI: 85.2–21.0 days) (log-rank χ^2^ = 5.120, *p* = 0.023).

There were too few MDS patients to accurately analyze the clinical activity of APVO436. The median survival for all seven patients was 151 days. Of the seven MDS patients enrolled, six were evaluable for response, and they had SD without a significant hematological improvement as their best overall response (Table S13). The times to progression intervals were 104 days, >106 days, 138 days, >147 days, 211 days, and 321 days for the six patients. Of these, three achieved a marrow CR (UPN23 from cohort 6A: pretreatment BM blast percentage: 7.5% with 10% marrow cellularity; C2D1 posttreatment BM blast percentage 2.4% with 20% marrow cellularity; UPN32 from cohort 7: pretreatment BM blast percentage: 11.3% with 20–30% marrow cellularity; C2D1 posttreatment BM blast percentage: 0% with 50% marrow cellularity; UPN39 from cohort 9: pretreatment BM blast percentage: 8.2% with 15% marrow cellularity; C2D1 posttreatment BM blast percentage: 2% with 20% marrow cellularity) (Table S13). Despite the very small patient numbers, this activity signal in MDS patients also warrants further clinical investigation.

## 4. Discussion

The greatest challenge in the field of AML therapy is achieving deep CRs and long-term leukemia-free survival in R/R AML patients [1,2,3,4,5,6,7,8,9,10,11,12,13,14,15,16]. Despite the fact that we have gained new insights and a better understanding of cell-intrinsic drivers of AML and new drugs have been developed for targeting select somatic mutations, the majority of R/R AML patients die of leukemia [8,9,10,11,12,13,14,15,16]. In patients at second or subsequent relapse, the median overall survival is ≤3 months, warranting the development and testing of novel therapies aimed at overcoming the drug resistance at relapse. While high allele frequencies of certain somatic mutations may provide an opportunity for precision medicines, there is no consensus on a single re-induction regimen, and evidence-based treatment guidelines generally recommend enrollment of such patients in a clinical trial or empiric use of one of the many cytotoxic re-induction regimens in patients not eligible for applicable clinical research protocols.

T cell redirecting bispecific antibodies represent a promising new class of potential anti-leukemia drugs [41,42,43,44,45,46]. In this phase 1B study, APVO436 exhibited a favorable safety profile, and its MTD was not reached at a dose level of 240 µg/cycle. Both prolonged SD and two CRs (evolved from PRs) were observed as early evidence of clinical efficacy in R/R AML patients when APVO436 was administered as weekly intravenous infusions at the 0.2 mcg/kg RP2D level (viz. flat dose levels of 12 or 18 µg) that corresponded to 30% of the highest safe dose level that was not associated with DLT in this study. Of the nine R/R AML/MDS patients treated at RP2D, one AML achieved a prolonged SD with time to progression of 238 days (UPN20, Table 4), two AML patients achieved a PR that deepened to a CR (UPN 21 and UPN28, Table 4), and one MDS patient achieved a marrow CR (UPN23, Table S13). Two patients had PD, and the two remaining patients had short SD as their best overall responses. An additional patient from cohort 7 had a complete clearance of peripheral blasts and a >50% reduction of BM blasts followed by sustained SD. These results are reminiscent of the partial and complete tumor regressions initially reported for blinatumomab at a flat dose of 15 µg [42]. It is also noteworthy that three of six evaluable R/R MDS patients achieved a marrow CR. Furthermore, this study provides the first clinical insights regarding the safety profile of APVO436 in R/R AML patients. Some patients treated with APVO436 experienced CRS and neurotoxicity. However, these AEs were medically manageable, and APVO436 could be administered safely to heavily pre-treated R/R AML and MDS patients, including those with advanced age. Drug-related myelosuppression was not observed. This favorable early safety and clinical activity profile of APVO436 in R/R AML and MDS patients warrants further clinical investigation.

Of the 34 relapsed AML patients evaluable for surrogate response measurements, 8 had clinically meaningful stabilization of their leukemia or a CR. Seven of these eight had failed 2–4 prior lines of anti-AML therapy, and one patient had relapsed after frontline therapy with Venetoclax plus Decitabine. The median OS was >300 days for the 8 R/R AML patients with a favorable response (prolonged SD and PRs/CRs). Five of the eight patients remained alive at 110, 124, 323, 352, and 395 days. The OS of these eight patients was significantly better than the survival outcome of the total population as well as non-responders. It is noteworthy that in mouse xenograft models of human AML, APVO436 exhibited anti-leukemic activity at HED levels of ≥0.08 mcg/kg with maximal activity obtained at HED of 0.4 mcg/kg [32]. In agreement with the preclinical proof-of-concept data, CRs were observed as best overall responses to APVO436 as a single agent at the anticipated sub-MTD clinical dose levels of 0.15–0.19 mcg/kg (UPN28 in cohort 6B, 12 mcg flat dose = ≈0.2 mcg/kg (BW: 80.3 kg); UPN21 in cohort 6A, 18 mcg flat dose = ≈0.2 mcg/kg (BW: 94.8 kg)). The off-the-shelf availability of APVO436, combined with its single agent anti-leukemic activity, favorable safety profile, and ease of administration not requiring continuous infusion but weekly short infusions (over 4 h after cycle 1) that can be administered in outpatient settings, makes it an attractive option as a bispecific T cell engager against AML.

A new study with five parallel-enrolling cohorts has been designed as a part 2 expansion phase of the AML study 5001 to further evaluate the tolerability and efficacy of APVO436 in poor prognosis AML patients when administered as monotherapy consolidation therapy, maintenance therapy for MRD positivity in remission with or without oral azacitidine, or as part of a multi-modality induction therapy in both newly diagnosed patients and patients in first relapse (Clinicaltrial.gov identifier: NCT03647800). We hypothesize that the use of APVO436 in patients whose immune system has not been subjected to several rounds of lymphotoxic chemotherapy will maximize the likelihood of clinically meaningful anti-leukemic activity against CD123-expressing AML blasts and leukemic stem cells. We are optimistic about those cohorts of the recently activated five-cohort expansion study that will explore the use of APVO436 in first remission after standard induction chemotherapy and for MRD-positive disease. There are also two novel cohorts using APVO436 in combination with salvage chemotherapy in R/R AML patients or Venetoclax and Azacitidine in fit patients with adverse risk disease (frontline and first relapse), with the goal to target and eliminate residual leukemic stem cells and prevent the emergence of drug resistance.

A major challenge in using classic bispecific T cell engagers (BiTEs) such as the CD33xCD3 bispecific antibody AMG330 are their short half-lives, owing to their lack of an Fc domain, requiring continuous intravenous infusion, reminiscent of the pharmacokinetics of the prototype BiTE, blinatumomab [41,42]. A new version of AMG330 with an Fc domain, AMG673, was also reported but—unlike APVO436—it was associated with a very high incidence of CRS: CRS was reported in 63% of 38 AML patients, with 18% grade 3 or higher events (Clinicaltrial.gov identifier: NCT03224819). Further, there were no CRs in any of the patients who received AMG673 at dose levels ranging from 5 to 110 mcg [43]. Flotetuzumab (MGD006) is a bispecific, dual-affinity re-targeting (DART) antibody reactive with both CD3 antigen on T cells and CD123 antigen on AML cells [44]. It is noteworthy that the short half-life of Flotetuzumab, reminiscent of BiTEs, requires IV infusions according to an intermittent 4 days on/3 days off schedule or continuous 7-day infusion [44]. By comparison, APVO436, due to its larger size and Fc domain, has a prolonged half-life and can be given via short IV infusions and weekly, thereby reducing the treatment burden for patients, their families, and treating physicians/nurses. On the other hand, bispecific antibodies with shorter half-lives might have a practical advantage of rapid elimination of the antibody from the system after its cessation in case on a serious toxicity.

Flotetuzumab exhibited promising single agent activity in therapy-refractory AML patients with primary induction failure as well as patients with an early first relapse [44] While Flotetuzumab exhibited anti-leukemic activity in AML patients with primary induction failure (PIF), it did not appear to be very active in relapsed AML patients who failed after being in remission post standard induction chemotherapy (Clinicaltrial.gov identifier: NCT02152956). This raises the possibility that Flotetuzumab may depend on target AML cells being actively damaged by chemotherapy in order to cause their destruction via T cell engagement. It has been proposed that immune-infiltrated, IFN-γ-dominant TME of PIF patients makes them more likely to respond to immunotherapy with bispecific antibodies [45]. Other CD123 x CD3 bispecific antibodies in early phase clinical trials in patients with R/R AML include SAR440334 (NCT03594955), a T cell-engaging multispecific monoclonal antibody, and JNJ-63709178 (NCT02715011), a humanized DuoBody. APVO436 has not yet been evaluated in PIF patients, and the expansion study that will examine its clinical benefit for PIF patients with refractory AML in one of its five cohorts has recently been activated (Clinicaltrial.gov identifier: NCT03647800). The ability of APVO436 to induce CRs in multiply relapsed AML patients demonstrated that the clinical activity of this bispecific antibody is not limited to patients with PIF, which appears to be a favorable differentiator to Flotetuzumab.

CD123 antigen is expressed not only on AML cells but also on CD34^+^ human hematopoietic stem cell and myeloid progenitor cell populations [21,47,48,49,50]. IMGN632 is an antibody drug conjugate (ADC) targeting CD123 on leukemia cells and carrying a covalently linked DNA alkylating agent as a payload. IMGN632 exhibited promising clinical activity in both BPDCN and AML patients with complete remissions achieved after monotherapy [51,52]. Of 66 evaluable AML patients, 3 (4.5%) achieved a CR [51]. Severe neutropenia was the most common adverse event, and VOD was also reported as a side effect [51]. IMGN632 is being evaluated in combination with Venetoclax and Azacitidine in patients with MRD^+^ remission (Clinicaltrial.gov identifier: NCT04401748) [52]. Another CD123-targeting biotherapeutic agent associated with severe neutropenia is the human IL3 fusion toxin Tagraxofusb (SL-401) [53,54], which was approved by the FDA in 2018 for treatment of blastic plasmacytoid dendritic cell neoplasm (BPDCN), being found to be not very active in AML at the MTD of 12.5 mcg/kg/day (Clinicaltrial.gov identifier: NCT02270463). The clinical success of Tagraxofusp is hampered by acquired deficiencies in the diphthamide synthesis pathway, leading to DPH1 deficiency/downregulation in AML patients causing resistance of leukemic blast cells to the truncated diphtheria toxin domain of the fusion toxin [53,54,55]. While no drug-related myelosuppression was reported in the current phase 1B study at the tested dose levels up to 60 mcg (≈0.7 mcg/kg), further clinical evaluation is needed to fully evaluate the effects of APVO436 on normal hematopoietic stem cell and myeloid progenitor cell populations, especially when it is used in combination with myelotoxic drugs. Huang et al. demonstrated that CD123 is expressed on myeloid progenitors but not on erythroid progenitor cells [55]. Therefore, the cause of the APVO436-related anemia that was observed in 2 of the 46 patients, both in cohort 4, is likely not an on-target toxicity to CD123-expressing immature erythroid cells. Future studies will study the incidence of this rare adverse event and explore the possibility of an immune-mediated intravascular hemolysis as a possible cause along with the benefit of steroids as a possible remedy. Additionally, we will examine the blood trombopoietin (TPO) levels in patients who develop thrombocytopenia in an attempt to discriminate between thrombocytopenia due to increased platelet destruction and decreased platelet production.

An aberrant production of the pro-inflammatory cytokines IL-6 and TNF-α is observed in AML patients [56]. Furthermore, high level CD123 expression has been detected in the lungs, raising the possibility of an on-target pulmonary toxicity for CD123-targeting biotherapeutics [57]. Therefore, risk mitigation strategies aimed at reducing the severity of CRS and CRS-associated pulmonary injury are particularly important to maximize patient safety for patients receiving CD123 targeting bispecific antibodies. All patients treated with Flotetuzumab were reported to develop mild-to-moderate (grade ≤2) CRS [44]. Vibecotamab (XmAb14045) is another bispecific antibody targeting both CD123 and CD3. A total of 104 patients with AML were treated at dosages from 0.003 to 12.0 µg/kg. There were two CRs (1.9%) and three CRi (2.9%). As with Flotetuzumab, CRS was the most common adverse event after treatment with Vibecotamab, occurring in 58% of the patients [46]. By comparison, only 10 of 46 patients treated with APVO 436 developed CRS. Cytokine profiling with measurement of serum levels of the proinflammatory cytokines interleukin-5 (IL-5), IL-6, interleukin-10 (IL-10), interleukin-17A (IL-17A), interferon-gamma (IFN-g), monocyte chemoattractant protein 1 (MCP-1), and tumor necrosis factor alpha (TNF-a) in patients who developed CRS after CD3-engaging bispecific antibody APVO436 indicates that the predominant cytokine in this inflammatory cytokine response is IL-6 [58]. The administration of Tocilizumab (antibody against IL-6:IL-6R) or Siltuximab (antibody against IL-6) at standard doses with or without dexamethasone rendered APVO436-associated severe CRS manageable and rapidly transient consistent with our current understanding of the IL-6 receptor signaling [59] and the standard management of this class-specific complication [27,28,29].

Older patients with newly diagnosed AML respond poorly to standard induction chemotherapy and have a poor survival. For newly diagnosed older AML patients, new treatment regimens have been developed in recent years, such the combination of the BCL-2 homology 3 (BH3)-mimetic compound; Venetoclax with HMA (e.g., azacitidine/AZA and decitabine/DAC); or the combination of the Hedgehog pathway inhibitor Glasdegib with LDARAC, both of which showed significant clinical activity with reduction in the risk of death in randomized phase II clinical trials [3,4,11,59]. Older patients with relapsed AML have a dismal prognosis and are in urgent need for new salvage treatment strategies for their chemo-resistant leukemia. Many of these patients are not transplant eligible due to age- and disease-related comorbidities/frailty as well as cumulative organ toxicity from previous chemotherapy. APVO436 was generally well tolerated in the older adults with relapsed AML with manageable toxicity and a promising benefit to risk profile. There was a trend towards a higher age for patients who had a favorable response with seven of eight (87.5%) responders, being in the ≥65-years poor prognosis age category with adverse cytogenetic features. The median age among the eight patients with favorable responses was 74.5 years, whereas the median age among non-responders was 66 years. Pending further validation in a larger patient population, these early findings indicate that APVO436 may have clinical impact potential as a salvage regimen for older patients with R/R AML. Notably, Venetoclax has recently been shown to augment T cell effector function by increased production of reactive oxygen species, and Azacitidine has been shown to enhance sensitivity of AML cells to cytotoxic T cells by activating the STING pathway [60]. These observations provide a compelling rationale for combining APVO436 with Venetoclax, Azacitidine, or both for the treatment of newly diagnosed high-risk or R/R AML patients. A new clinical study (Clinicaltrials.gov identifier: NCT04973618) has been initiated to evaluate the tolerability and efficacy of APVO436 in combination with Venetoclax plus Azacitidine in AML patients ≥75 years of age or those who are over 60 years of age and unfit for intensive chemotherapy or HSCT. Importantly, activating mutations of the FLT3 receptor are common mutations in AML, and FLT3 has been shown to be mutated and/or upregulated in CD123^+^ AML [61,62,63,64]. Therefore, bispecific antibodies alone or in combination with FLT3 inhibitors (e.g., midostaurin) may be useful in designing patient-tailored and risk-adjusted strategies aimed at improving the quality of remission and thereby reduce the risk of relapse.

## 5. Conclusions

Our phase 1B clinical study of APVO436 provided new information that this novel bispecific antibody is generally well tolerated by R/R AML patients with an MTD of greater than 60 mcg. Of the 34 R/R AML patients, evaluable for surrogate response measurements, 8 had clinically meaningful stabilization of their leukemia. Two patients developed first a PR and then a CR. Among the six evaluable R/R MDS patients, three achieved a marrow CR. Median OS was 338.5 days for the eight responding/stable R/R AML patients. APVO436 was generally well tolerated in the older adults, with R/R AML with manageable toxicity related to CSR/IRRs. The safety, feasibility, and preliminary clinical activity of APVO436 in R/R AML and MDS deserves further clinical investigation, and combination therapy cohorts as well as consolidation/MRD therapy cohorts are now underway for AML patients in an expansion study.

## Figures and Tables

**Table 1:** Patient characteristics, demographic features, and APVO436 exposure for safety population (N = 46).

Diagnosis	
AML	39 (84.8%)
Primary AML	26 (56.5%)
Secondary (s)-AML	9 (19.6%)
Treatment related (t)-AML	4 (8.7%)
MDS	7 (15.2%)
**Age** (years)	
Mean ± SE	65.4 ± 2.0
Median	69
Range	18–82
**Sex**	
Female	22 (47.8%)
Male	24 (52.2%)
**Ethnic origin**	
Caucasian, not Hispanic or Latino	34 (73.9%)
Caucasian, Hispanic or Latino	6 (13.0)
Black or African American	3 (6.5%)
Hispanic or Latino	1 (2.2%)
Asian	2 (4.3%)
**Prior # of chemotherapy regimens**	
1	9 (19.6%)
2	14 (30.4%)
3	6 (13.0%)
≥4	16 (34.8%)
Range	1–8
Not reported	1 (2.2%)
Mean ± SE (median)	3.2 ± 0.3 (2.5)
**Number of APVO436 treatments** Mean ± SE (median)	11 ± 2 (7)

**Table 2:** Incidence of APVO436-related grade 3–5 AEs occurring in R/R AML/MDS patients treated on phase 1B dose escalation study 5001.

MedDRA SOC MedDRA PT	Cohorts												Total
	CH1 (N = 4)	CH2 (N = 3)	CH3 (N = 3)	CH4 (N = 6)	CH5 (N = 3)	CH6A (N = 6)	CH6B (N = 3)	CH7 (N = 4)	CH8 (N = 6)	CH9 (N = 3)	CH10 (N = 4)	Other (N = 1)	N = 46 *n* (%)
**Blood and lymphatic system disorders**													
**Anemia**	**0**	**0**	**0**	**2**	**0**	**0**	**0**	**0**	**0**	**0**	**0**	**0**	**2 (4.3%)**
Grade 3	0	0	0	2	0	0	0	0	0	0	0	0	2 (4.3%)
**Cardiac disorders**													
**Acute myocardial infarction**	**0**	**0**	**0**	**0**	**0**	**0**	**0**	**0**	**1**	**0**	**0**	**0**	**1 (2.2%)**
Grade 3	0	0	0	0	0	0	0	0	1	0	0	0	1 (2.2%)
**Gastrointestinal disorders**													
**Diarrhea**	**0**	**0**	**0**	**0**	**0**	**1**	**0**	**0**	**0**	**0**	**0**	**0**	**1 (2.2%)**
Grade 3	0	0	0	0	0	1	0	0	0	0	0	0	1 (2.2%)
**Nausea**	**0**	**0**	**0**	**1**	**0**	**0**	**0**	**0**	**0**	**0**	**0**	**0**	**1 (2.2%)**
Grade 3	0	0	0	1	0	0	0	0	0	0	0	0	1 (2.2%)
**Vomiting**	**0**	**0**	**0**	**0**	**0**	**1**	**0**	**0**	**0**	**0**	**0**	**0**	**1 (2.2%)**
Grade 3	0	0	0	0	0	1	0	0	0	0	0	0	1 (2.2%)
**General disorders and administration site conditions**													
**Asthenia**	**0**	**0**	**0**	**0**	**0**	**1**	**0**	**0**	**0**	**0**	**0**	**0**	**1 (2.2%)**
Grade 3	0	0	0	0	0	1	0	0	0	0	0	0	1 (2.2%)
**Chills**	**1**	**0**	**0**	**0**	**0**	**0**	**0**	**0**	**0**	**0**	**0**	**0**	**1 (2.2%)**
Grade 3	1	0	0	0	0	0	0	0	0	0	0	0	1 (2.2%)
**Fatigue**	**0**	**0**	**0**	**1**	**0**	**0**	**0**	**0**	**0**	**0**	**0**	**0**	**1 (2.2%)**
Grade 3	0	0	0	1	0	0	0	0	0	0	0	0	1 (2.2%)
**Immune system disorders**													
**Cytokine release syndrome**	**1**	**0**	**0**	**1**	**0**	**1**	**0**	**0**	**1**	**0**	**0**	**0**	**4 (8.7%)**
Grade 3	1	0	0	0	0	1	0	0	1	0	0	0	3 (6.5%)
Grade 4	0	0	0	1	0	0	0	0	0	0	0	0	1 (2.2%)
**Infections and infestations**													
**Sepsis**	**0**	**0**	**0**	**0**	**0**	**1**	**0**	**0**	**0**	**0**	**0**	**0**	**1 (2.2%)**
Grade 3	0	0	0	0	0	1	0	0	0	0	0	0	1 (2.2%)
**Injury, poisoning and procedural complications**													
**Infusion related reaction**	**1**	**0**	**1**	**0**	**0**	**0**	**0**	**0**	**0**	**0**	**0**	**0**	**2 (4.3%)**
Grade 3	1	0	1	0	0	0	0	0	0	0	0	0	2 (4.3%)
**Investigations**													
**Platelet count decreased**	**0**	**0**	**0**	**1**	**0**	**0**	**0**	**0**	**0**	**0**	**0**	**0**	**1 (2.2%)**
Grade 4	0	0	0	1	0	0	0	0	0	0	0	0	1 (2.2%)
**Metabolism and nutrition disorders**													
**Fluid overload**	**0**	**0**	**0**	**0**	**0**	**0**	**0**	**0**	**1**	**0**	**0**	**0**	**1 (2.2%)**
Grade 3	0	0	0	0	0	0	0	0	1	0	0	0	1 (2.2%)
**Hyperglycemia**	**0**	**0**	**0**	**1**	**0**	**0**	**0**	**0**	**0**	**0**	**0**	**0**	**1 (2.2%)**
Grade 3	0	0	0	1	0	0	0	0	0	0	0	0	1 (2.2%)
**Tumor lysis syndrome**	**0**	**0**	**0**	**1**	**0**	**0**	**0**	**0**	**0**	**0**	**0**	**0**	**1 (2.2%)**
Grade 3	0	0	0	1	0	0	0	0	0	0	0	0	1 (2.2%)
**Renal and urinary disorders**													
**Acute kidney injury**	**0**	**0**	**0**	**2**	**0**	**0**	**0**	**0**	**0**	**0**	**0**	**0**	**2 (4.3%)**
Grade 3	0	0	0	1	0	0	0	0	0	0	0	0	1 (2.2%)
Grade 5	0	0	0	1	0	0	0	0	0	0	0	0	1 (2.2%)
**Respiratory, thoracic, and mediastinal disorders**													
**Dyspnea**	**1**	**0**	**0**	**0**	**0**	**0**	**0**	**0**	**0**	**0**	**0**	**0**	**1 (2.2%)**
Grade 3	1	0	0	0	0	0	0	0	0	0	0	0	1 (2.2%)
**Hypoxia**	**0**	**0**	**0**	**0**	**0**	**1**	**0**	**0**	**0**	**0**	**0**	**0**	**1 (2.2%)**
Grade 3	0	0	0	0	0	1	0	0	0	0	0	0	1 (2.2%)
**Acute hypoxemic resp. failure**	**0**	**0**	**0**	**1**	**0**	**0**	**0**	**0**	**0**	**0**	**0**	**0**	**1 (2.2%)**
Grade 4	0	0	0	1	0	0	0	0	0	0	0	0	1 (2.2%)
**Pulmonary infiltrates**	**0**	**0**	**0**	**0**	**0**	**1**	**0**	**0**	**0**	**0**	**0**	**0**	**1 (2.2%)**
Grade 3	0	0	0	0	0	1	0	0	0	0	0	0	1 (2.2%)
**Pleural effusion**	**0**	**0**	**0**	**1**	**0**	**0**	**0**	**0**	**0**	**0**	**0**	**0**	**1 (2.2%)**
Grade 3	0	0	0	1	0	0	0	0	0	0	0	0	1 (2.2%)
**Vascular disorders**													
**Hypotension**	**1**	**0**	**0**	**0**	**0**	**0**	**0**	**0**	**0**	**0**	**0**	**0**	**1 (2.2%)**
Grade 3	1	0	0	0	0	0	0	0	0	0	0	0	1 (2.2%)
**Shock**	**0**	**0**	**0**	**1**	**0**	**0**	**0**	**0**	**0**	**0**	**0**	**0**	**1 (2.2%)**
Grade 4	0	0	0	1	0	0	0	0	0	0	0	0	1 (2.2%)

Note: When the same event was reported twice for the same patient, it was only counted once, and the highest grade (worst grade) was captured. CH: cohort.

**Table 3:** Listing of all APVO436-related serious adverse events (SAEs).

Patient No.	Cohort#	SAE Reported Term (CTCAE Grade)	Start Date (CxDx); # of Days from ICF	End Date (# of Days from ICF	Total Duration of the SAE (Days)	SAE Outcome	Changes to Drug Dose or Schedule	SUSAR (Yes/No)
UPN02	1	CRS (3)	C6D1; 148	155	8	Resolved	DPD	Yes
		Rigors (3)	C6D1; 148	150	3	Resolved	DPD	No
		Chills (3)	C6D1; 148	150	3	Resolved	DPD	No
		Dyspnea (3)	C6D1; 148	150	3	Resolved	DPD	No
		Hypotension (3)	C6D1; 148	150	3	Resolved	DPD	No
UPN04	1	IRR (1)—fever (3)	C1D8; 14	16	3	Resolved	None	Yes
UPN12	4	Acute renal failure (5) (complication of CRS [2])	C2D1; 55	55	1	Fatal	DPD	Yes
		CRS (2)	C2D1; 43	NA	>12	NR	DD	No
UPN14	4	CRS (1)	C1D3; 10	13	4	Resolved	DD	Yes
UPN16	4	CRS (4)	C1D5; 19	24	6	Partially Resolved	DPD	Yes
		Respiratory failure—acute (4)	C1D5; 19	21	3	Resolved	DPD	Yes
UPN17	5	IRR (1)	C3D1; 65	67	3	Resolved	None	No
UPN20	6A	Sepsis (3)	C6D15; 165	169	5	Resolved	TI	Yes
UPN22	6A	CRS (3)	C1D3; 6	15	10	Resolved	DR/DD	No
		Pulmonary edema	C1D3;6	15	10	resolved	DD	No
		Hypoxia intermittent (3)	C1D3; 6	10	5	Resolved	DD	No
		Worsening dyspnea (2)	C1D3; 6	15	10	Resolved	DD	No
		Pulmonary infiltrates (3)	C1D3; 6	15	10	Resolved	DD	No
UPN24	6A	Generalized weakness (3)	C1D5; 11	38	28	Resolved	DD	Yes
UPN30	7	IRR (2)	C2D15; 56	59	4	Resolved	TI	No
UPN31	7	CRS (2)	C5D1; 120	121	2	Resolved	TI	No
		Rigors (2)	C5D1; 120	120	1	Resolved	None	No
UPN38	8	N-STEMI 2° to CRS (3)	C1D1; 5	6	2	Resolved	TI	No
		Fluid overload (3)	C1D11; 21	23	3	Resolved	None	No
		Fever (2)	C1D1; 5	5	1	Resolved	TI	No
		Hypotension (3)	C1D1; 5	5	1	Resolved	TI	No
		Rigor (2)	C1D1; 5	5	1	Resolved	TI	No
		CRS (3)	C1D1; 5	6	2	Resolved	TI	No
UPN46	NA	Neurotoxicity (1)	C1D1; 10	11	2	Resolved	DPD	Yes

In UPN16 CRS was complicated by grade 3 pleural effusion, grade 4 acute hypoxemic respiratory failure, and grade 3 acute kidney injury (AKI). UPN46 was also assigned to treatment with APVO436 according to an exploratory dose-intense daily regimen but was taken off the study after developing grade 1 neurotoxicity with transient confusion during the first infusion (6 mcg intended dose level; actual dose received: 1.5 mcg). TI: temporarily interrupted (including interruption of infusion or discontinuation of infusion for the day of the AE); DPD: drug permanently discontinued; DD: next dose delayed; DR: dose reduced; CRS: cytokine release syndrome.

**Table 4:** Patient characteristics and demographic features of APVO436-treated AML patients with favorable responses.

								BM Involvement				Treatment Outcome			
Patient No.	Cohort	# of APVO 436 Doses	Diagnosis	Age/Sex/Race	BMI/CRS/Neurotoxicity	Previous Therapies (Number: List)	Cellularity	Percent Myeloblasts	Karyotype/Mutations	Time between Failure of Last AML Therapy (PD) and C1D1 on APVO436	Best Overall Response	Time to Best Overall Response (days)	Time to Progression (days)	Time to Death or Hospice	Survival Status at Last FU
UPN02	1	21	t-AML	75/M/C	29.7/Yes/No	4: TCP/ATRA; 5AZA; Exp.X2	90	12	Unknown/ KRAS, TET2, U2AF1	7	SD	32	188	188	D
UPN17	5	12	1° AML	65/M/A	26.5/No/No	3: IDAC; HiDAC; ME	10–30	10–15	Unknown/ND	29	SD	33	87	194	D
UPN20	6A	33	1° AML	73/F/C	18.3/Yes/Yes	2: Vyxeos; AZA + Venetoclax	70–80	15	del(20q)	15	SD	36	238	>395	A
UPN21	6A	24	1° AML	74/M/C	31.7/Yes/No	3: 7 + 3; Exp.x2	10	30	46, XY/ IDH1, IDH2, ZRSR2	15	PR CR	58 113	169	>323	A
UPN28	6B	40	1° AML	76/M/C	24.7/No/Yes	1: Decitabine + Venetoclax	20	29	−7, del(5q)/TP53, NF1	39	PR CR	31 92	288	>352	A
UPN31	7	28	1° AML	78/F/C	20.4/Yes/Yes	4:AZA; TCP; Pevonedistat; TRA/Triretinoin; LD-ARAC	100	78	46, XX/None	14	SD + PBBC-C + >50% BMB reduction	36	211	282	D
UPN42	10	9	1° AML	47/M/B	25/No/No	3: FLU/CTX; FLAG-IDA; Decitabine	20–50	4	t(2;15)/ NF1, RUNX1, GATA2, IKZF1	25	SD	75	>110	>110	A
UPN44	10	13	1° AML	82/M/C	28.6/No/No	2: AZA; AZA + Venetoclax	20	50	del(12p)/None	18	SD	41	>124	>124	A

M: male; C: Caucasian; B: Black/African American; t-AML: treatment-related AML; 1° AML: primary AML; BMI: body mass index; CRS: cytokine release syndrome; PD: progressive disease; A: alive; D: dead; ND: not determined; SD: stable disease; PR: partial remission; CR: complete remission; TCP: tranylcycpromine; BMB: BM blast. UPN31 had a WBC of 5.3 × 10^9^/L pretreatment. For an estimated 2 L blood volume, the 21% leukemic blasts in circulation corresponded to a total of 2.3 x10^9^ AML blasts. The peripheral blast percentage was 21% pretreatment, 14% on C2D22, 2% on C3D1, 1% on C4D1, and 0% on C4D22 (day 113 after informed consent). The clearance of circulating leukemia cells corresponded to >9-log reduction of the blood leukemic load by APVO436 therapy. See text for further details.

## Data Availability

Will individual participant data be available (including data dictionaries)? Yes. What data in particular will be shared? Individual participant data that underlie the results reported in this article, after de-identification (text, tables, figures, and appendices). What other documents will be available? Study protocol. When will data be available (start and end dates)? Beginning 3 months and ending 5 years following article publication. With whom? Researchers who provide a methodologically sound proposal. For what types of analyses? To achieve aims in the approved proposal.

## Supplementary Materials

The following are available online at https://www.mdpi.com/article/10.3390/cancers13164113/s1, Supplemental Methods, Supplemental Safety Data, and Supplemental figure and tables, including: Figure S1: Survival Outcome of AML/MDS Patients, Table S1: R/R AML/MDS Patients Treated Weekly with APVO436 in Different Dose Cohorts of APVO436-5001 Phase 1B Study (N = 46), Table S2: Cohort-Specific APVO436 Exposure Data, Table S3: Incidence of all treatment-emergent AE by MedDRA PT and worst CTCAE Grade occurring in patients treated with APVO436 in Study 5001 regardless of any relationship with the study drug APVO436, Table S4: Incidence of all APVO436-related AE by MedDRA PT and worst CTCAE Grade occurring in patients treated with APVO436 in Study 5001, Table S5: Incidence of adverse events by MedDRA PT and worst CTCAE Grade ≥3 severity occurring in patients treated with APVO436 in Study 5001 regardless of any relationship with the study drug APVO436, Table S6: Listing of all APVO436-related Grade ≥3 AE occurring in patients treated with APVO436 in Study 5001, Table S7: Febrile neutropenia (FBN) in AML/MDS patients treated with APVO436 in Study 5001, Table S8: Incidence of all APVO436-related SAE by MedDRA PT and worst CTCAE Grade occurring in patients treated with APVO436 in Study, Table S9: Predictors of APVO436-Related SAE and Lack of SAE Impact on Disease Progression or Survival Time, Table S10: APVO436-related neurotoxicity occurring in patients treated with APVO436 in Phase 1B Study 5001, Table S11: Predictors of APVO436-related Neurotoxicity and Its Lack of Impact on Overall Survival, Table S12: Predictors of Favorable Response and Its Impact on Time to Progression of Leukemia, Table S13: Patient Characteristics, Demographic Features, and Treatment Outcome for APVO436-Treated MDS Patients.
