# Supplementary material for: A Clinical Phase 1B Study of the CD3xCD123 Bispecific Antibody APVO436 in Patients with Relapsed/Refractory Acute Myeloid Leukemia or Myelodysplastic Syndrome
Source: Cancers (Basel). 2021 Aug 15;13(16):4113. doi: 10.3390/cancers13164113 (PMC8394899; doi:10.3390/cancers13164113)
Supplement: Supplementary file 1 [file cancers-13-04113-s001.zip › cancers-1326876-supplementary.pdf]

SUPPLEMENTAL INFORMATION

SUPPLEMENTAL TABLES AND FIGURES

Tables S1 – S11 provide detailed safety data. **Table S12** and **Table S13** provide information related to the single agent activity of APVO436. **Figure S1** shows the survival outcome of the patient population.

SUPPLEMENTAL METHODS

Patients and Patient Disposition. 58 patients were screened; 12 patients were screen failures, and the remaining 46 patients were enrolled in the study. Eligibility for the study required patients had to be ≥ 18 years of age with histologically confirmed relapsed or refractory AML and not be a candidate for intensive chemotherapy or allogeneic hematopoietic stem cell transplantation (HSCT). Alternatively, patients had to have relapsed or refractory myelodysplasia (MDS) with > 5% blasts in the marrow or any blasts in the peripheral blood. MDS patients were required to have failed prior treatment with standard hypomethylating agents (HMA) such as azacitidine or decitabine. CD123 expression on AML/MDS cells was not a requirement for inclusion. Eligibility required adequate performance status with an Eastern Cooperative Oncology Group (ECOG) performance score of 0, 1, or 2. Adequate kidney and liver function as well as coagulation were required. Patients with acute promyelocytic leukemia (APL) with t(15;17), CNS leukemia, other active systemic malignancies, graft versus host disease (GvHD) or autoimmune disorders requiring immunosuppressive therapy, or uncontrolled active infections were excluded. Exclusion criteria included symptomatic congestive heart failure ≥ Class III (New York Heart Association Functional Classification), uncontrolled hypertension, unstable angina pectoris, myocardial infarction within previous 6 months, clinically significant arrhythmias not controlled by medication, as well as uncontrolled metabolic disorders such as hypercalcemia. Patients were required to not have any residual unresolved Grade >1 AEs according to National Cancer Institute Common Terminology Criteria for Adverse Events [NCI CTCAE] that resulted from previous standard or experimental treatments and were clinically significant. White blood cells (WBC) had to be ≤ 25,000 cells/mm3 and patients were allowed to receive hydroxyurea to bring their WBC count down prior to and during the first cycle of treatment with study drug if necessary.

Details of Clinical Study Design and Study Conduct

This first-in-human (FIH) clinical trial of APVO436 was designed as a multiple-dose Phase 1B dose escalation study in patients with relapsed AML and high-risk myelodysplasia (MDS) (Clinicaltrial.gov identifier: NCT03647800). It was performed under IND 135552 at the following

10 centers in the US as an open-label study sponsored by Aptevo Therapeutics: (1) Greenville Health System, Institute for Translational Oncology Research, Greenville, South Carolina, United States, 29605; (2) The University of Kansas Clinical Research Center, Westwood, Kansas, United States, 66205; (3) Sylvester Comprehensive Cancer Center, University of Miami, Miami, Florida, United States, 33136; (4) University of Utah, Huntsman Cancer Institute, Salt Lake City, Utah, United States, 84112; (5) The Ohio State University Wexner Medical Center/James Cancer Hospital ; Columbus, Ohio, United States, 43210; (6) Roswell Park Cancer Institute; Buffalo, New York, United States, 14263; (7) Fred Hutchinson Cancer Research Center; Seattle, Washington, United States, 98109; (8) University of Texas Southwestern Medical Center, Dallas, Texas, United States, 75390; (9) University of Florida College of Medicine, Gainesville, Florida, United States, 32610; (10) University of California, San Francisco Medical Center, San Francisco, California, United States, 94143.

The study protocol was approved by the WCG-Central Institutional Review Board (IRB) (OHRP/FDA registration number: IRB00000533) and the local IRB at participating centers (OHRP/FDA registration/Federalwide Assurance numbers: FWA00001380, IRB00005621, FWA00003745, FWA00006731, FWA00001920, FWA00005087, FWA00000068). The Central IRB-approved study/protocol number was 20181730. The study was performed in compliance with the International Conference on Harmonization (ICH) guidelines for Good Clinical Practice (ICHE6/GCP). Each patient provided a written informed consent (ICF) prior to enrollment. The main purpose of the study was to determine the recommended Phase 2 dose (RP2D) level of APVO436. Patients in Cohorts 1-10 received APVO436 intravenously (IV) for six 28-day cycles. Patients were permitted to stay on study drug for up to a total of 6 cycles until disease progression, unacceptable toxicities, or withdrawal of consent. Patients who do not achieve at least SD by the end of Cycle 2 will be discontinued from the study drug. Patients with evidence of clinical benefit (i.e., CRi, PR, SD) at the end of Cycle 6 in the absence of unacceptable toxicity may also continue on study for up to 36 total cycles at the discretion of Investigator provided the patient did not experience a dose limiting toxicity (DLT) and there was no other contraindication.

The starting dose in Cohort 1 was 0.3 mcg (~0.005 mcg/kg for a 60-kg patient) which was the Minimum Anticipated Biological Effect Level (MABEL) based on T-cell activation assays. The assigned weekly target dose levels for Cohorts 2-10 were 1 mcg, 3 mcg, 9 mcg, 18 mcg (Cohort 6A), 12 mcg (Cohort 6B), 24 mcg, 36 mcg, 48 mcg, and 60 mcg, respectively. A 3+3 design was used to guide the dose escalation. All patients in a specific dose cohort were required to complete one cycle of therapy and an evaluation for AEs and DLTs during a 28-day DLT observation period, and the safety data reviewed with the Investigators and the Safety Review Committee (SRC) before enrollment in the next dose cohort could begin. For the first 2 patients within each dose cohort, administration of the first dose was separated by a minimum of 36

hours for risk mitigation. For further risk mitigation and maximized patient safety, all patients were hospitalized for safety monitoring for a minimum of 72 hours after the start of the first dose in Cycle 1 in Cohorts 1 to 7 and for 36 hours in Cohorts 8 to 10. The first dose of APVO436 was administered over 20 to 24 hours. In addition, any time there was a dose increase, APVO436 was administered over 20 to 24 hours. If necessary, to manage or prevent any AE, and in particular IRR or CRS, any dose infusion may be slowed and/or interrupted, with the administration time extended up to 72 hours.

All patients were pre-medicated with acetaminophen, an antihistamine and a corticosteroid (Dexamethasone) before every dose. All 3 premedications were initially required, but dexamethasone was optional after the sixth dose (i.e., after C2D15) as long as the patient had not experienced any IRRs or CRS. Patients were observed for 24 hours after the first increased dose has been infused. The observation period after the second dose was at least 4 hours. The observation period after the third and subsequent doses was at least 2 hours. After 2 cycles at the increased dose, if the patient had no infusion-related AEs, then the observation period could be decreased to 1 hour following completion of the infusion.

The SRC and Investigators reviewed the adverse events (AE) and laboratory data and dose reductions and/or dose delays were instituted if deemed necessary for patient safety.
For each patient in Cohorts 1 through 4, APVO436 was infused over approximately 20 to 24 hours for the first dose (C1D1), over 8 hours for the second dose (C1D8), over 6 hours for the third dose (C1D15) and over 4 hours for all subsequent doses (C1D22 and onwards). Beginning in Cohort 5, and for all cohorts going forward, stepped dosing was introduced to mitigate against the development of infusion related reactions (IRR) and cytokine release syndrome (CRS). If necessary, to manage or prevent any AE, and in particular IRR or CRS, any dose infusion was allowed to be slowed and/or interrupted, with the administration time extended up to 72 hours to maximize patient safety. If an infusion was extended past 60 hours, then the patient was observed for 12 hours after the infusion was completed. The investigators based decisions to dose de-escalate, hold, or discontinue APVO436 for an individual patient using the NCI Common Terminology Criteria for Adverse Events (CTCAE) Version 5.0 toxicity grades. Dose escalation was performed according to **Table S2**.

Cohort 6 consisted of two dose cohorts that were conducted concurrently. Cohort 6a was the same as Cohort 5 except doses ≥ 4th were 18 mcg rather than 12 mcg; Cohort 6b tested whether the 2nd dose during the step-up dosing can be escalated to 12 mcg rather than 9 mcg as used in Cohort 5. The results from Cohort 6a and 6b informed which dosing regimen was selected for Cohort 7 to 10.

The maximum tolerated dose (MTD) was defined as the highest dose at which no more than 1 of 6 patients within a cohort experience a DLT. The MTD was predefined as one dose below the dose at which ≥ 2 of 3 patients or ≥ 2 of 6 patients experience a DLT any time during the DLT observation period.

Response according to the International Working Group (IWG) 2006 criteria were used for assessment of MDS patients. Standard European LeukemiaNet 2017 criteria were used for response assessments in AML patients (Doehner). The clinical activity endpoints for AML were composite overall response (composite CR plus MLFS and PR) and CR as defined by the ELN 2017 criteria.

A Dose-Limiting Toxicity (DLT) was defined as any of the following AEs (per CTCAE or as specified below) that occurs within 28 days of the first dose of APVO436. In general, a DLT was an AE or abnormal laboratory value that was assessed as at least possibly related to APVO436 and unrelated to either underlying disease, disease progression, or concurrent illness. The DLTs were assessed and defined in 3 distinct categories:

1. Cytokine Release Syndrome (CRS) DLT:
   a. Any Grade ≥ 3 CRS toxicity that occurs within 28 days of the first dose of APVO436 and is related, likely related or possibly related to APVO436 infusion and unrelated to either underlying disease, disease progression, or concurrent illness, and doesn't resolve within 24 hours of maximal medical support
2. Hematologic DLT:
   a. ANC < 500 cells/mm^3 (500 cells/uL) by Day 42 after the last dose in Cycle 1 in the setting of a BM showing < 5% blasts without evidence of persistent cytogenetic abnormalities or immunophenotypic evidence of leukemia. Myelosuppression was considered secondary to leukemia if there was cytogenetic or immunophenotypic evidence of disease. Beyond the provisional assignment by Day 42, patients who meet the above hematologic DLT criteria and continue to have neutrophil counts < 500/uL were required to have a repeat BM biopsy by Day 56. During this additional 2-week period, a patient who continued to meet the hematologic DLT criteria would be considered to have a definitive DLT. However, patients who have evidence of ANC recovery > 500 cells/mm^3 (500 cells/uL) by Day 56 would not be counted as a DLT event.
3. Non-hematologic DLT:
   a. Any non-CRS or non-hematological Grade ≥ 3 toxicity, related, likely related or possibly related to APVO436 and unrelated to either underlying disease, disease progression or concurrent illness is a DLT.
   b. Nausea, vomiting, or diarrhea Grade ≥ 3 that is not controlled by maximal medical palliation and results in hospitalization, TPN, or tube feeding is a DLT.

c. Any IRR (e.g., fever, chills, nausea, vomiting, headache, hypotension) Grade ≥ 3 toxicity occurring within one day of APVO436 infusion that does not resolve within 24 hours with maximal medical support is a DLT.
d. Any CNS toxicity that does not resolve within 72 hours and is related, likely related or possibly related to APVO436, and is unrelated to either underlying disease, disease progression or concurrent illness, is a DLT.
e. A single Grade 3 or 4 laboratory abnormality not associated with clinical sequelae was not considered a DLT, unless it was confirmed with a repeat test and was considered clinically significant and related, likely related or possibly related to APVO436, and unrelated to either underlying disease, disease progression or concurrent illness.
f. Any Grade 4 organ toxicity is a DLT.
g. Toxicities that are expected and related to the underlying disease of AML or MDS or to disease progression were not considered a DLT

Patients were required to meet one of the following criteria to be evaluable for dose-escalation decisions to the next dose cohort:

1. Has received all 4 doses of APVO436 and has completed Cycle 1, Day 28 without a DLT; or
2. Has experienced a DLT prior to completing Cycle 1, Day 28.

If a patient did not meet either of these criteria and did not complete the DLT observation period because of progressive disease, withdrawal of consent, or another reason besides toxicity, then the patient was not evaluable for determination of MTD or cohort dose-escalation decisions, and was to be replaced in that cohort. However, any patient receiving any amount of APVO436 was included in the Safety Population.

SUPPLEMENTAL SAFETY DATA

The list of all treatment-emergent AEs irrespective of their relatedness to the study drug APVO436 is provided in **Table S3**. The most common AEs observed in >20% of the patients were pyrexia (15 of 46 patients, 32.6%), infusion related reaction (IRR) (13 of 46 patients, 28.3%), peripheral edema (12 of 46 patients, 26.1%), anemia (11 of 46 patients, 23.9%), cytokine release syndrome (CRS) (10 of 46 patients, 21.7%), febrile neutropenia (10 of 46 patients, 21.7%), and chills (10 of 46 patients, 21.7%). The incidence of all APVO436-related AEs of any grade are shown in **Table S4**. The most common APVO436-related AEs were IRR occurring in 13 (28.3%) patients and CRS occurring in 10 (21.7%). The list of all treatment-emergent Grade ≥3 AEs irrespective of their relatedness to the study drug APVO436 is provided in **Table S5**, which includes febrile neutropenia (12 episodes in 10 of 46 patients,

21.7%), anemia (7 of 46 patients, 15.2%), hyperglycemia (6 of 46 patients, 13.0%), sepsis (5 of 46 patients, 10.9%), and platelet count decreased (5 of 46 patients, 10.9%).

SUPPLEMENTAL TABLES AND FIGURES

Table S1 : R/R AML/MDS Patients Treated Weekly with APVO436 in Different Dose Cohorts of APVO436-5001 Phase 1B Study (N=46)*										
**Cohort 1 N=4 0.3 µg**	**Cohort 2 N=3 1 µg**	**Cohort 3 N=3 3 µg**	**Cohort 4 N=6 9 µg**	**Cohort 5 N=3 12 µg**	**Cohort 6A N=6 18 µg**	**Cohort 6B N=3 12 µg**	**Cohort 7 N=4 24 µg**	**Cohort 8 N=6 36 µg**	**Cohort 9 N=3 48 µg**	**Cohort 10 N=4 60 µg**
UPN01	UPN05	UPN08	UPN11	UPN17	UPN20	UPN26	UPN29	UPN33	UPN 39	UPN42
UPN02	UPN06	UPN09	UPN12	UPN18	UPN21	UPN27	UPN30	UPN34	UPN40	UPN43
UPN03	UPN07	UPN10	UPN13	UPN19	UPN22	UPN28	UPN31	UPN35	UPN41	UPN44
UPN04			UPN14		UPN23		UPN32	UPN36		UPN45
			UPN15		UPN24			UPN37		
			UPN16		UPN25			UPN38		

Assigned dose(s) in each cohort:
Cohort 1: 0.3 mcg/week;
Cohort 2: 1 mcg/week;
Cohort 3: 3 mcg/week;
Cohort 4: 9 mcg/week;
Cohort 5: Week 1: 6 mcg, Week 2: 9 mcg, Week ≥3: 12 mcg;
Cohort 6A: Week 1: 6 mcg, Week 2: 9 mcg, Week 3: 12 mcg, Week ≥3: 18 mcg;
Cohort 6B: Week 1: 6 mcg, Week ≥2: 12 mcg;
Cohort 7: Week 1: 6 mcg, Week 2: 12 mcg, Week 3: 18 mcg, Week ≥3: 24 mcg;
Cohort 8: Week 1: 6 mcg, Week 2: 12 mcg, Week 3: 18 mcg, Week ≥3: 36 mcg;
Cohort 9: Week 1: 6 mcg, Week 2: 12 mcg, Week 3: 18 mcg, Week ≥3: 48 mcg;
Cohort 10: Week 1: 6 mcg, Week 2: 12 mcg, Week 3: 18 mcg, Week ≥3: 60 mcg

*A total of 58 patients were screened and there were 12 screen failures. 45 patients received APVO436 according to a once a week iv. Administration schedule. An additional patient not listed on this table (UPN46) was also assigned to treatment with APVO436 according to an exploratory dose-intense daily regimen, but was taken off the study after developing Grade 1 neurotoxicity with transient confusion during the first infusion (6 mcg intended dose level; actual dose received: 1.5 mcg). Therefore, the total number of patients treated with APVO436 was 46.

UPN38 in Cohort 8 was originally assigned to treatment with APVO436 according to an exploratory dose-intense daily regimen, received 6 mcg of APVO436 on day 1 and developed Grade 3 CRS during the second infusion. The cumulative dose infused was 8.5 mcg. CRS was complicated by non-ST elevation myocardial infarction (N-STEMI). Patient's CRS and cardiac function fully recovered and he was switched to the weekly regimen and entered into Cohort 8.

Table S2: Cohort-Specific APVO436 Exposure Data

Cohort #	Assigned Dose Level mcg	Cumulative Dose (mcg)	Cumulative Dose/kg BW	Highest Single Dose (ng/kg)
**Cohort 1**	0.3	Mean: 3.4±1.9<br>Median: 1.7<br>Range: 1.2-9.1	Mean (ng/kg): 46.3±22.1<br>Median (ng/kg): 32.0<br>Range: 12.0-109.0	Mean: 4.5±0.7<br>Median: 4<br>Range: 3-6
**Cohort 2**	1.0	Mean: 5.3±1.3<br>Median: 4.0<br>Range: 4.0-8.0	Mean (ng/kg): 54.7±11.2<br>Median (ng/kg): 45.0<br>Range (ng/kg): 42.0-77.0	Mean: 10.7±0.4<br>Median: 4<br>Range: 3-6
**Cohort 3**	3.0	Mean: 18.0±6.0<br>Median: 12.0<br>Range: 12.0-30.0	Mean (ng/kg): 200.0±57.7<br>Median (ng/kg): 200.0<br>Range (ng/kg): 100.0-300.0	Mean: 36.0±4.9<br>Median: 32<br>Range: 31-44
**Cohort 4**	9.0	Mean: 97.5±58.3<br>Median: 45.0<br>Range: 9.0-387.0	Mean (mcg/kg): 1.3±0.8<br>Median (mcg/kg): 0.8<br>Range (mcg/kg): 0.1-5.1	Mean: 126.7±9.0<br>Median: 118.0<br>Range: 108-159
**Cohort 5**	12.0<br>(6-9-12-12)	Mean: 75.0±25.0<br>Median: 63.0<br>Range: 39.0-123.0	Mean (mcg/kg): 0.9±0.3<br>Median (mcg/kg): 0.7<br>Range (mcg/kg): 0.5-1.5	Mean: 141.3±5.1<br>Median: 133<br>Range: 133-146
**Cohort 6A**	18.0<br>(6-9-12-18)	Mean: 212.5±83.2<br>Median: 174.0<br>Range: 6.0-471.0	Mean (mcg/kg): 3.3±1.6<br>Median (mcg/kg): 2.4<br>Range (mcg/kg): 0.1-10.4	Mean: 239±51<br>Median: 199<br>Range: 83-396
**Cohort 6B**	12.0<br>(6-12-12-12)	Mean: 194.0±140.2<br>Median: 66.0<br>Range: 42.0-474.0	Mean (mcg/kg): 2.6±1.6<br>Median (mcg/kg): 1.3<br>Range (mcg/kg): 0.7-5.9	Mean: 210.3±45.4<br>Median: 149<br>Range: 149-277
**Cohort 6A+6B Combined**	12.0-18.0	Mean: 206.3±67.3<br>Median: 66.0<br>Range: 6.0-474.0	Mean (mcg/kg): 3.1±1.1<br>Median (mcg/kg): 1.3<br>Range (mcg/kg): 0.1-10.4	Mean: 229.5±34.1<br>Median: 222<br>Range: 83-396
**Cohort 7**	24.0<br>(6-12-18-24)	Mean: 267.0±129.9<br>Median: 198.0<br>Range: 36.0-636.0	Mean (mcg/kg): 4.3±2.5<br>Median (mcg/kg): 2.4	Mean: 335.5±44.5<br>Median: 285<br>Range: 260-444

			Range (mcg/kg): 0.7-11.8	
**Cohort 8**	36.0 (6-12-18-36)	Mean: 271.4±135.2 Median: 162.0 Range: 36.0-936.0	Mean (mcg/kg): 3.8±1.9 Median (mcg/kg): 2.3 Range (ng/kg): 0.5-13.1	Mean: 512.7±53.8 Median: 528 Range: 289-615
**Cohort 9**	48.0 (6-12-18-48)	Mean: 621.7±244.5 Median: 495.0 Range: 276.0-1094.0	Mean (mcg/kg): 6.7±1.7 Median (mcg/kg): 5.7 Range (ng/kg): 4.3-10.1	Mean: 651.7±209.1 Median: 522 Range: 443-990
**Cohort 10**	60.0 (6-12-18-60)	Mean: 475.5±136.5 Median: 465.0 Range: 156.0-816.0	Mean (mcg/kg): 5.8±1.7 Median (mcg/kg): 5.8 Range (ng/kg): 1.7-9.8	Mean: 715.3±28.3 Median: 710 Range: 662-779

Table S3: Incidence of all treatment-emergent AE by MedDRA PT and worst CTCAE Grade occurring in patients treated with APVO436 in Study 5001 regardless of any relationship with the study drug APVO436

MedDRA SOC<br>MedDRA PT	Cohorts<br>Cohort 1 (N=4)	Cohort 2 (N=3)	Cohort 3 (N=3)	Cohort 4 (N=6)	Cohort 5 (N=3)	Cohort 6A (N=6)	Cohort 6B (N=3)	Cohort 7 (N=4)	Cohort 8 (N=6)	Cohort 9 (N=3)	Cohort 10 (N=4)	Other (N=1)	Total<br>N = 46<br>n (%)
**Blood and lymphatic system disorders**													
Anemia	1	1	0	3	0	1	1	1	1	1	1	0	11 (23.9%)
Disseminated intravascular coagulation	0	0	0	1	0	0	0	0	0	0	0	0	1 (2.2%)
Febrile neutropenia	1	1	0	2	2	2	1	0	2	0	0	0	12 (26.1%)
Leukocytosis	1	0	0	0	0	0	0	0	0	1	0	0	2 (4.3%)
Leukopenia	0	0	0	0	0	0	0	1	0	0	0	0	1 (2.2%)
Neutropenia	0	0	0	0	0	0	0	1	0	0	0	0	1 (2.2%)
Thrombocytopenia	0	0	0	0	0	0	0	1	1	0	0	0	2 (4.3%)
**Cardiac disorders**													
Acute myocardial infarction	0	0	0	0	0	0	0	0	1	0	0	0	1 (2.2%)
Atrial enlargement	0	0	0	0	0	0	1	0	0	0	0	0	1 (2.2%)
Atrial fibrillation	0	0	0	0	0	1	0	0	0	0	0	0	1 (2.2%)
Diastolic dysfunction	0	0	0	1	0	0	0	0	0	0	0	0	1 (2.2%)
Palpitations	0	0	0	0	0	0	0	1	0	0	0	0	1 (2.2%)
Sinus bradycardia	0	0	0	1	0	0	0	0	0	0	0	0	1 (2.2%)
Sinus tachycardia	0	0	0	1	0	0	1	0	0	0	0	0	2 (4.3%)
Supraventricular tachycardia	0	0	0	0	0	1	0	0	0	0	0	0	1 (2.2%)
Tachycardia	0	0	0	2	0	1	0	1	1	1	0	0	6 (13.0%)
**Ear and labyrinth disorders**													
Ear pain	0	0	0	1	0	0	0	0	0	0	0	0	1 (2.2%)
Excessive cerumen production	0	0	0	1	0	0	0	0	0	0	0	0	1 (2.2%)
**Eye disorders**													
Conjunctival hyperemia	0	0	0	0	0	0	0	1	0	0	0	0	1 (2.2%)
Dry eye	0	0	1	0	0	0	0	0	0	0	0	0	1 (2.2%)
Ocular hyperemia	0	0	0	0	0	0	0	0	0	1	0	0	1 (2.2%)
Periorbital edema	0	0	0	0	0	0	1	0	0	0	0	0	1 (2.2%)
Sudden visual loss	0	0	0	0	0	0	0	0	0	0	1	0	1 (2.2%)
**Gastrointestinal disorders**													
Abdominal distension	0	0	1	0	0	0	0	1	0	0	0	0	2 (4.3%)
Abdominal pain	0	0	1	1	0	1	0	1	0	0	0	0	4 (8.7%)
Abdominal pain lower	0	0	0	0	0	0	0	0	1	0	0	0	1 (2.2%)
Angina bullosa hemorrhagic	0	0	0	0	0	0	0	1	0	0	0	0	1 (2.2%)
Constipation	0	0	1	0	0	1	0	0	1	1	1	0	5 (10.9%)
Diarrhea	1	0	2	3	1	3	0	2	1	1	0	0	14 (30.4%)
Dry mouth	0	0	0	1	0	0	0	0	0	0	1	0	2 (4.3%)
Dysphagia	0	0	0	0	0	1	0	0	0	0	0	0	1 (2.2%)
Gastritis	0	0	1	0	0	0	0	0	0	0	0	0	1 (2.2%)
Gingival bleeding	1	0	0	0	0	1	0	0	0	0	0	0	2 (4.3%)
Gingival pain	0	0	0	0	0	0	0	0	0	0	1	0	1 (2.2%)
Hemorrhoids	0	0	0	0	1	0	0	0	0	0	0	0	1 (2.2%)
Lower gastrointestinal hemorrhage	0	0	0	0	0	1	0	0	0	0	0	0	1 (2.2%)

MedDRA SOC<br>MedDRA PT	Cohorts												Total
	Cohort 1 (N=4)	Cohort 2 (N=3)	Cohort 3 (N=3)	Cohort 4 (N=6)	Cohort 5 (N=3)	Cohort 6A (N=6)	Cohort 6B (N=3)	Cohort 7 (N=4)	Cohort 8 (N=6)	Cohort 9 (N=3)	Cohort 10 (N=4)	Other (N=1)	N = 46 n (%)
Mouth hemorrhage	0	0	0	0	0	0	2	0	0	0	0	0	2 (4.3%)
Nausea	0	1	1	1	0	2	0	1	0	1	0	0	7 (15.2%)
Oral mucosal blistering	0	0	0	0	0	0	0	1	0	0	0	0	1 (2.2%)
Oral pain	0	0	0	0	0	1	0	0	0	0	1	0	2 (4.3%)
Stomatitis	1	0	0	0	1	0	0	0	0	0	0	0	2 (4.3%)
Tongue hematoma	1	0	0	0	0	0	0	0	0	0	0	0	1 (2.2%)
Toothache	0	0	0	0	0	0	0	0	0	1	0	0	1 (2.2%)
Vomiting	0	0	1	0	0	1	0	1	0	0	0	0	3 (6.5%)
**General disorders and administration site conditions**													
Asthenia	0	0	0	0	0	1	0	0	0	1	0	0	2 (4.3%)
Catheter site erythema	1	0	0	0	0	0	0	1	1	1	0	0	4 (8.7%)
Catheter site edema	0	0	0	0	0	0	0	0	1	0	0	0	1 (2.2%)
Catheter site pain	0	0	0	0	0	1	0	0	1	0	0	0	2 (4.3%)
Catheter site pruritus	1	0	0	0	0	0	0	0	0	0	0	0	1 (2.2%)
Catheter site vesicles	0	0	0	0	0	0	0	0	1	0	0	0	1 (2.2%)
Chest discomfort	0	0	0	0	0	1	0	0	0	0	0	0	1 (2.2%)
Chills	1	1	0	2	1	1	0	2	0	1	1	0	10 (21.7%)
Fatigue	0	1	0	3	1	3	2	1	0	0	1	0	12 (26.1%)
Gait disturbance	0	0	0	0	0	2	0	0	0	0	0	0	2 (4.3%)
Generalized edema	0	0	0	1	0	0	0	0	0	0	1	0	2 (4.3%)
Injection site induration	0	0	0	1	0	0	0	0	0	0	0	0	1 (2.2%)
Malaise	1	0	0	0	0	0	0	0	0	0	0	0	1 (2.2%)
Mucosal inflammation	0	0	0	0	0	0	1	0	0	0	0	0	1 (2.2%)
Edema peripheral	1	1	0	5	0	0	1	1	2	1	0	0	12 (26.1%)
Pain	0	0	0	1	0	0	0	0	0	0	0	0	1 (2.2%)
Peripheral swelling	0	0	0	0	0	1	0	0	1	0	0	0	2 (4.3%)
Pyrexia	2	0	3	2	0	2	1	2	2	1	0	0	15 (32.6%)
**Hepatobiliary disorders**													
Hyperbilirubinemia	0	0	0	0	0	0	0	1	0	0	0	0	1 (2.2%)
**Immune system disorders**													
Cytokine release syndrome	1	0	0	3	0	3	0	1	2	0	0	0	10 (21.7%)
**Infections and infestations**													
Abscess oral	0	0	0	1	0	0	0	0	0	0	0	0	1 (2.2%)
Anal abscess	0	0	0	0	1	0	0	0	0	0	0	0	1 (2.2%)
Bronchitis	1	0	0	0	0	0	0	0	0	0	0	0	1 (2.2%)
Candida infection	0	0	0	0	0	2	0	0	0	0	0	0	2 (4.3%)
Catheter site cellulitis	0	0	0	0	0	0	0	0	1	0	0	0	1 (2.2%)
Cellulitis	1	0	0	2	0	2	0	0	0	0	0	0	5 (10.9%)
Clostridium difficile colitis	0	0	0	0	0	0	0	0	1	0	0	0	1 (2.2%)
Corona virus infection	0	0	0	0	0	0	0	0	0	0	1	0	1 (2.2%)
Enteritis infectious	0	0	0	1	0	0	0	0	0	0	0	0	1 (2.2%)
Localized infection	0	0	0	0	0	0	0	0	1	0	0	0	1 (2.2%)
Lower respiratory tract infection fungal	0	0	0	0	0	0	0	1	0	0	0	0	1 (2.2%)

MedDRA SOC<br>MedDRA PT	Cohorts												Total
	Cohort 1 (N=4)	Cohort 2 (N=3)	Cohort 3 (N=3)	Cohort 4 (N=6)	Cohort 5 (N=3)	Cohort 6A (N=6)	Cohort 6B (N=3)	Cohort 7 (N=4)	Cohort 8 (N=6)	Cohort 9 (N=3)	Cohort 10 (N=4)	Other (N=1)	N = 46<br>n (%)
Lung infection	0	0	0	1	0	0	0	0	0	0	0	0	1 (2.2%)
Oral herpes	0	0	0	1	0	0	0	0	0	0	0	0	1 (2.2%)
Pneumonia	0	0	0	0	0	1	0	0	0	0	0	0	1 (2.2%)
Sepsis	1	0	1	0	0	2	0	0	1	0	0	0	5 (10.9%)
Septic shock	0	0	0	0	0	1	0	0	0	0	0	0	1 (2.2%)
Sialadenitis	0	0	0	0	0	1	0	0	0	0	0	0	1 (2.2%)
Sinusitis	0	0	0	1	0	0	0	0	0	0	1	0	2 (4.3%)
Tooth infection	0	0	0	0	0	0	0	1	0	0	1	0	2 (4.3%)
Wound infection	0	0	0	1	0	0	0	0	0	0	0	0	1 (2.2%)
**Injury, poisoning and procedural complications**													
Clavicle fracture	0	0	0	1	0	0	0	0	0	0	0	0	1 (2.2%)
Contusion	0	0	0	1	0	1	1	1	0	0	1	0	5 (10.9%)
Fall	1	0	0	2	0	0	0	0	0	0	0	0	3 (6.5%)
Infusion related reaction	1	0	1	3	0	2	0	3	1	2	0	0	13 (28.3%)
Limb injury	1	0	0	0	0	0	0	0	0	0	0	0	1 (2.2%)
Rib fracture	0	0	0	1	0	0	0	0	0	0	0	0	1 (2.2%)
Subcutaneous hematoma	0	0	0	1	0	0	0	0	0	0	0	0	1 (2.2%)
Transfusion reaction	0	0	0	1	0	0	0	0	0	0	0	0	1 (2.2%)
**Investigations**													
Activated partial thromboplastin time prolonged	0	0	0	1	0	0	0	0	0	0	0	0	1 (2.2%)
Alanine aminotransferase increased	0	0	0	2	0	0	0	1	1	0	0	0	4 (8.7%)
Aspartate aminotransferase increased	0	0	0	2	0	0	0	0	1	0	0	0	3 (6.5%)
Blood alkaline phosphatase increased	0	0	0	1	0	0	0	0	1	0	1	0	3 (6.5%)
Blood bilirubin increased	0	0	0	0	0	0	0	0	1	0	1	0	2 (4.3%)
Blood creatinine increased	1	0	0	1	0	1	0	0	0	1	0	0	4 (8.7%)
Blood lactate dehydrogenase increased	0	0	0	0	0	1	0	0	1	0	1	0	3 (6.5%)
C-reactive protein increased	0	0	0	0	0	0	0	0	0	0	1	0	1 (2.2%)
Inflammatory marker increased	0	0	0	0	0	0	0	0	0	0	1	0	1 (2.2%)
International normalized ratio increased	0	0	1	1	0	0	0	0	0	0	1	0	3 (6.5%)
Lymphocyte count decreased	0	0	0	1	0	0	0	1	1	0	1	0	4 (8.7%)
Lymphocyte count increased	0	0	0	1	0	0	0	0	0	0	0	0	1 (2.2%)
Neutrophil count decreased	0	0	0	0	0	1	0	0	0	0	0	0	1 (2.2%)
Platelet count decreased	0	1	0	2	0	0	0	0	2	0	1	0	6 (13.0%)
Serum ferritin increased	0	0	0	1	0	0	0	0	0	0	0	0	1 (2.2%)
Troponin I increased	0	0	0	1	0	0	0	0	0	0	0	0	1 (2.2%)
Urine output decreased	0	0	0	1	0	0	0	0	0	0	0	0	1 (2.2%)
White blood cell count decreased	0	0	0	1	0	1	0	0	1	0	0	0	3 (6.5%)
White blood cell count increased	0	0	0	1	0	0	0	0	0	0	0	0	1 (2.2%)
**Metabolism and nutrition disorders**													
Decreased appetite	0	0	0	1	1	2	0	0	0	0	0	0	4 (8.7%)
Dehydration	0	0	0	0	0	1	0	0	0	0	0	0	1 (2.2%)
Diabetes mellitus	0	0	0	0	0	1	0	0	0	0	0	0	1 (2.2%)

MedDRA SOC<br>MedDRA PT	Cohorts												Total
	Cohort 1 (N=4)	Cohort 2 (N=3)	Cohort 3 (N=3)	Cohort 4 (N=6)	Cohort 5 (N=3)	Cohort 6A (N=6)	Cohort 6B (N=3)	Cohort 7 (N=4)	Cohort 8 (N=6)	Cohort 9 (N=3)	Cohort 10 (N=4)	Other (N=1)	N = 46 n (%)
Fluid overload	1	0	0	0	0	1	0	0	2	0	0	0	4 (8.7%)
Hyperglycemia	1	0	1	2	0	1	0	2	1	0	1	0	9 (19.6%)
Hyperkalemia	1	0	0	0	0	0	0	0	1	0	0	0	2 (4.3%)
Hyperlipidemia	0	0	1	0	0	0	0	0	0	0	0	0	1 (2.2%)
Hypomagnesaemia	0	0	0	1	0	0	0	0	0	0	1	0	2 (4.3%)
Hyperphosphatemia	0	0	1	0	0	0	0	0	0	0	0	0	1 (2.2%)
Hypertriglyceridemia	0	0	0	1	0	0	0	0	1	0	0	0	2 (4.3%)
Hyperuricemia	1	0	0	1	0	0	0	0	0	0	0	0	2 (4.3%)
Hypoalbuminemia	0	0	0	2	0	0	0	0	0	0	1	0	3 (6.5%)
Hypocalcemia	0	0	0	1	0	1	0	1	0	0	1	0	4 (8.7%)
Hypokalemia	1	1	0	3	2	1	0	1	1	0	0	0	10 (21.7%)
Hypomagnesaemia	0	0	1	1	1	1	0	0	1	0	2	0	7 (15.2%)
Hyponatremia	0	0	0	2	0	1	0	1	0	0	0	0	4 (8.7%)
Hypophosphatemia	0	0	0	0	0	2	0	0	0	0	1	0	3 (6.5%)
Lactic acidosis	0	0	0	0	0	0	1	0	0	0	0	0	1 (2.2%)
Tumor lysis syndrome	0	0	0	1	0	0	0	0	1	0	0	0	2 (4.3%)
**Musculoskeletal and connective tissue disorders**													
Arthralgia	0	1	0	1	0	0	0	1	0	0	2	0	5 (10.9%)
Back pain	0	0	0	0	0	1	0	0	0	0	1	0	2 (4.3%)
Bone pain	0	0	1	0	0	0	0	0	0	0	0	0	1 (2.2%)
Flank pain	0	0	0	1	0	0	0	0	0	0	0	0	1 (2.2%)
Joint swelling	0	0	0	0	0	0	0	0	1	0	0	0	1 (2.2%)
Limb discomfort	0	0	0	0	0	1	0	0	0	0	0	0	1 (2.2%)
Muscular weakness	0	0	0	0	1	0	0	0	0	0	1	0	2 (4.3%)
Musculoskeletal pain	0	0	0	0	0	1	0	1	0	0	0	0	2 (4.3%)
Myalgia	1	0	0	1	0	1	0	0	0	0	0	0	3 (6.5%)
Neck pain	0	0	0	0	0	0	0	0	0	0	1	0	1 (2.2%)
Pain in extremity	0	0	0	0	0	0	0	0	2	0	0	0	2 (4.3%)
**Nervous system disorders**													
Amnesia	0	0	0	0	0	0	0	1	0	0	0	1	2 (4.3%)
Aphasia	0	0	0	0	0	0	0	0	1	0	0	0	1 (2.2%)
Cerebral hemorrhage	1	0	0	0	0	0	0	0	0	0	0	0	1 (2.2%)
Cerebral ischemia	0	0	0	1	0	0	0	0	0	0	0	0	1 (2.2%)
Depressed level of consciousness	0	0	0	0	0	0	0	0	0	0	1	0	1 (2.2%)
Dizziness	0	0	0	3	0	2	0	0	0	0	0	0	5 (10.9%)
Dysgeusia	0	0	0	1	0	0	0	0	0	0	0	0	1 (2.2%)
Encephalopathy	0	0	0	0	0	1	0	0	0	0	0	0	1 (2.2%)
Headache	0	0	0	1	0	0	1	0	0	1	0	0	3 (6.5%)
Lethargy	0	0	0	0	0	2	0	0	0	0	0	0	2 (4.3%)
Memory impairment	0	0	0	0	0	0	0	1	0	0	0	0	1 (2.2%)
Neuropathy peripheral	0	0	0	0	0	0	0	1	0	0	0	0	1 (2.2%)
Neurotoxicity	0	0	0	0	0	0	0	0	0	0	0	1	1 (2.2%)
Peripheral sensory neuropathy	0	0	0	1	0	0	1	0	0	0	0	0	2 (4.3%)

MedDRA SOC<br>MedDRA PT	Cohorts												Total
	Cohort 1 (N=4)	Cohort 2 (N=3)	Cohort 3 (N=3)	Cohort 4 (N=6)	Cohort 5 (N=3)	Cohort 6A (N=6)	Cohort 6B (N=3)	Cohort 7 (N=4)	Cohort 8 (N=6)	Cohort 9 (N=3)	Cohort 10 (N=4)	Other (N=1)	N = 46<br>n (%)
Tremor	0	0	0	1	0	0	0	0	0	0	0	0	1 (2.2%)
**Psychiatric disorders**													
Anxiety	0	0	0	0	0	0	0	0	0	1	0	0	1 (2.2%)
Confusional state	0	0	0	1	0	1	0	1	1	0	2	1	7 (15.2%)
Delirium	0	0	0	0	0	2	0	0	0	0	0	0	2 (4.3%)
Depression	1	0	0	0	0	1	0	0	0	1	0	0	3 (6.5%)
Hallucination	0	0	0	0	0	0	0	1	0	0	0	0	1 (2.2%)
Insomnia	1	0	0	1	0	0	1	0	0	0	1	0	4 (8.7%)
**Renal and urinary disorders**													
Acute kidney injury	1	0	0	2	0	1	0	0	0	0	0	0	4 (8.7%)
Dysuria	0	0	0	0	0	1	0	0	1	0	0	0	2 (4.3%)
Hematuria	0	0	0	1	0	0	0	0	0	0	0	0	1 (2.2%)
Nocturia	0	0	0	1	0	0	0	0	0	0	0	0	1 (2.2%)
Proteinuria	0	0	0	1	0	0	0	0	0	0	0	0	1 (2.2%)
Urinary retention	0	0	0	0	0	2	0	0	0	0	0	0	2 (4.3%)
**Reproductive system and breast disorders**													
Vaginal discharge	0	0	0	0	0	0	0	0	0	0	1	0	1 (2.2%)
**Respiratory, thoracic and mediastinal disorders**													
Acute respiratory failure	0	0	0	1	0	2	0	0	0	0	0	0	3 (6.5%)
Cough	1	0	0	1	0	1	0	1	0	0	1	0	5 (10.9%)
Dysphonia	0	0	0	0	0	1	0	0	0	0	0	0	1 (2.2%)
Dyspnea	1	1	0	1	1	2	0	1	1	0	0	0	7 (15.2%)
Dyspnea exertional	0	0	0	0	0	0	1	0	0	0	0	0	1 (2.2%)
Epistaxis	1	0	0	0	0	0	0	0	1	0	1	0	3 (6.5%)
Hiccups	0	0	0	0	0	1	0	0	0	0	0	0	1 (2.2%)
Hypoxia	1	0	0	1	0	2	1	0	0	0	1	0	6 (13.0%)
Oropharyngeal pain	1	0	0	1	0	1	0	0	0	0	0	0	3 (6.5%)
Orthopnea	0	0	0	1	0	0	0	0	0	0	0	0	1 (2.2%)
Pleural effusion	0	0	0	1	0	0	0	0	0	0	0	0	1 (2.2%)
Pulmonary edema	0	0	1	1	0	1	0	0	0	0	0	0	3 (6.5%)
Pulmonary infiltrates	0	0	0	0	0	1	0	0	0	0	0	0	1 (2.2%)
Rales	1	0	0	0	0	0	0	0	0	0	0	0	1 (2.2%)
Respiratory failure	1	0	0	0	0	0	0	0	0	0	0	0	1 (2.2%)
Rhinorrhea	0	0	0	0	0	0	0	1	0	0	0	0	1 (2.2%)
Tachypnea	0	0	0	0	0	1	0	0	0	0	0	0	1 (2.2%)
Wheezing	0	0	0	1	0	0	0	0	0	0	0	0	1 (2.2%)
**Skin and subcutaneous tissue disorders**													
Dry skin	0	0	0	0	0	0	0	1	0	0	0	0	1 (2.2%)
Erythema	0	0	0	0	0	2	0	0	0	0	0	0	2 (4.3%)
Hyperhidrosis	0	0	0	1	0	0	0	0	0	0	1	0	2 (4.3%)
Night sweats	0	1	1	0	0	0	0	0	0	0	0	0	2 (4.3%)
Petechiae	0	0	0	1	0	0	0	0	0	0	0	0	1 (2.2%)
Pigmentation disorder	0	0	0	0	0	0	0	0	0	1	0	0	1 (2.2%)

MedDRA SOC<br>MedDRA PT	Cohorts												Total
	Cohort 1 (N=4)	Cohort 2 (N=3)	Cohort 3 (N=3)	Cohort 4 (N=6)	Cohort 5 (N=3)	Cohort 6A (N=6)	Cohort 6B (N=3)	Cohort 7 (N=4)	Cohort 8 (N=6)	Cohort 9 (N=3)	Cohort 10 (N=4)	Other (N=1)	N = 46 n (%)
Pruritus	1	0	0	1	0	0	0	0	0	0	0	0	2 (4.3%)
Purpura	0	0	0	0	0	0	0	1	0	0	0	0	1 (2.2%)
Rash	1	0	0	0	0	0	0	0	2	0	0	0	3 (6.5%)
Rash maculo-papular	0	1	0	0	0	0	0	0	0	0	0	0	1 (2.2%)
Skin exfoliation	0	0	0	0	0	0	0	1	1	0	0	0	2 (4.3%)
Skin induration	0	0	0	1	0	0	0	0	0	0	0	0	1 (2.2%)
Skin ulcer	0	0	0	0	1	0	0	0	0	0	0	0	1 (2.2%)
**Vascular disorders**													
Deep vein thrombosis	0	0	0	0	0	1	0	0	0	0	0	0	1 (2.2%)
Flushing	1	0	0	1	0	3	0	1	0	0	0	0	6 (13.0%)
Hematoma	0	0	0	1	0	0	0	0	0	0	0	0	1 (2.2%)
Hot flush	0	1	0	0	0	0	0	0	0	0	0	0	1 (2.2%)
Hypertension	0	1	1	1	0	0	0	1	0	1	0	0	2 (4.3%)
Hypotension	2	0	1	2	0	1	1	1	0	1	0	0	9 (19.6%)
Shock	0	0	0	1	0	0	0	0	0	0	0	0	1 (2.2%)

Note: When the same event was reported twice for the same patient, it was only counted once and the highest grade (worst grade) was captured.

Table S4: Incidence of all APVO436-related AE by MedDRA PT and worst CTCAE Grade occurring in patients treated with APVO436 in Study 5001

MedDRA SOC<br>MedDRA PT	Cohort 1 (N=4)	Cohort 2 (N=3)	Cohort 3 (N=3)	Cohort 4 (N=6)	Cohort 5 (N=3)	Cohort 6A (N=6)	Cohort 6B (N=3)	Cohort 7 (N=4)	Cohort 8 (N=6)	Cohort 9 (N=3)	Cohort 10 (N=4)	Other (N=1)	Total N = 46 n (%)
**Blood and lymphatic system disorders**													
**Anemia**	**0**	**0**	**0**	**2**	**0**	**0**	**0**	**0**	**0**	**0**	**0**	**0**	**2 (4.3%)**
Grade 3	0	0	0	2	0	0	0	0	0	0	0	0	2 (4.3%)
**Disseminated intravascular coagulation**	**0**	**0**	**0**	**1**	**0**	**0**	**0**	**0**	**0**	**0**	**0**	**0**	**1 (2.2%)**
Grade 2	0	0	0	1	0	0	0	0	0	0	0	0	1 (2.2%)
**Cardiac disorders**	0	0	0	0	0	0	0	0	2	1	0	0	3 (6.5%)
**Acute myocardial infarction/ N-STEMI**	**0**	**0**	**0**	**0**	**0**	**0**	**0**	**0**	**1**	**0**	**0**	**0**	**1 (2.2%)**
Grade 3	0	0	0	0	0	0	0	0	1	0	0	0	1 (2.2%)
**Tachycardia**	**0**	**0**	**0**	**0**	**0**	**0**	**0**	**0**	**1**	**1**	**0**	**0**	**2 (4.3%)**
Grade 1	0	0	0	0	0	0	0	0	1	0	0	0	1 (2.2%)
Grade 2	0	0	0	0	0	0	0	0	0	1	0	0	1 (2.2%)
**Gastrointestinal disorders**													
**Abdominal pain**	**0**	**0**	**0**	**0**	**0**	**1**	**0**	**0**	**0**	**0**	**0**	**0**	**1 (2.2%)**
Grade 1	0	0	0	0	0	1	0	0	0	0	0	0	1 (2.2%)
**Diarrhea**	**1**	**0**	**0**	**2**	**0**	**1**	**0**	**0**	**1**	**0**	**0**	**0**	**5 (10.9%)**
Grade 1	1	0	0	2	0	0	0	0	1	0	0	0	4 (8.7%)
Grade 3	0	0	0	0	0	1	0	0	0	0	0	0	1 (2.2%)
**Dry mouth**	**0**	**0**	**0**	**1**	**0**	**0**	**0**	**0**	**0**	**0**	**0**	**0**	**1 (2.2%)**
Grade 2	0	0	0	1	0	0	0	0	0	0	0	0	1 (2.2%)
**Nausea**	**0**	**0**	**0**	**1**	**0**	**1**	**0**	**1**	**0**	**0**	**0**	**0**	**3 (6.5%)**
Grade 1	0	0	0	0	0	1	0	1	0	0	0	0	2 (4.3%)
Grade 3	0	0	0	1	0	0	0	0	0	0	0	0	1 (2.2%)
**Vomiting**	**0**	**0**	**0**	**0**	**0**	**1**	**0**	**0**	**0**	**0**	**0**	**0**	**1 (2.2%)**
Grade 3	0	0	0	0	0	1	0	0	0	0	0	0	1 (2.2%)

MedDRA SOC<br>MedDRA PT	Cohort 1<br>(N=4)	Cohort 2<br>(N=3)	Cohort 3<br>(N=3)	Cohort 4<br>(N=6)	Cohort 5<br>(N=3)	Cohort 6A<br>(N=6)	Cohort 6B<br>(N=3)	Cohort 7<br>(N=4)	Cohort 8<br>(N=6)	Cohort 9<br>(N=3)	Cohort 10<br>(N=4)	Other<br>(N=1)	Total<br>N = 46<br>n (%)
**General disorders and administration site conditions**													
**Asthenia**	**0**	**0**	**0**	**0**	**0**	**1**	**0**	**0**	**0**	**0**	**0**	**0**	**1 (2.2%)**
Grade 3	0	0	0	0	0	1	0	0	0	0	0	0	1 (2.2%)
**Chills**	**1**	**0**	**0**	**1**	**0**	**0**	**0**	**0**	**0**	**1**	**0**	**0**	**3 (6.5%)**
Grade 1	0	0	0	1	0	0	0	0	0	0	0	0	1 (2.2%)
Grade 2	0	0	0	0	0	0	0	0	0	1	0	0	1 (2.2%)
Grade 3	1	0	0	0	0	0	0	0	0	0	0	0	1 (2.2%)
**Fatigue**	**0**	**0**	**0**	**3**	**0**	**1**	**1**	**0**	**0**	**0**	**0**	**0**	**5 (10.9%)**
Grade 1	0	0	0	0	0	0	1	0	0	0	0	0	1 (2.2%)
Grade 2	0	0	0	2	0	1	0	0	0	0	0	0	3 (6.5%)
Grade 3	0	0	0	1	0	0	0	0	0	0	0	0	1 (2.2%)
**Gait disturbance**	**0**	**0**	**0**	**0**	**0**	**1**	**0**	**0**	**0**	**0**	**0**	**0**	**1 (2.2%)**
Grade 1	0	0	0	0	0	1	0	0	0	0	0	0	1 (2.2%)
**Malaise**	**1**	**0**	**0**	**0**	**0**	**0**	**0**	**0**	**0**	**0**	**0**	**0**	**1 (2.2%)**
Grade 1	1	0	0	0	0	0	0	0	0	0	0	0	1 (2.2%)
**Edema peripheral**	**0**	**0**	**0**	**1**	**0**	**0**	**0**	**0**	**0**	**0**	**0**	**0**	**1 (2.2%)**
Grade 2	0	0	0	1	0	0	0	0	0	0	0	0	1 (2.2%)
**Pyrexia**	**1**	**0**	**0**	**0**	**0**	**1**	**1**	**0**	**0**	**1**	**0**	**0**	**4 (8.7%)**
Grade 1	1	0	0	0	0	1	1	0	0	1	0	0	4 (8.7%)
**Immune system disorders**													
**Cytokine release syndrome**	**1**	**0**	**0**	**3**	**0**	**3**	**0**	**1**	**2**	**0**	**0**	**0**	**10 (21.7%)**
Grade 1	0	0	0	1	0	2	0	0	1	0	0	0	4 (8.7%)
Grade 2	0	0	0	1	0	0	0	1	0	0	0	0	1 (2.2%)
Grade 3	1	0	0	0	0	1	0	0	1	0	0	0	4 (8.7%)
Grade 4	0	0	0	1	0	0	0	0	0	0	0	0	1 (2.2%)
**Infusion related reaction (IRR)**	**0**	**0**	**1**	**3**	**0**	**2**	**0**	**3**	**1**	**2**	**1**	**0**	13(28.3.%)
Grade 1	0	0	0	3	0	1	0	2	1	0	1	0	8(17.4%)

MedDRA SOC<br>MedDRA PT	Cohort 1<br>(N=4)	Cohort 2<br>(N=3)	Cohort 3<br>(N=3)	Cohort 4<br>(N=6)	Cohort 5<br>(N=3)	Cohort 6A<br>(N=6)	Cohort 6B<br>(N=3)	Cohort 7<br>(N=4)	Cohort 8<br>(N=6)	Cohort 9<br>(N=3)	Cohort 10<br>(N=4)	Other<br>(N=1)	Total<br>N = 46<br>n (%)
Grade 2	0	0	0	0	0	1	0	1	0	2	0	0	4 (8.7%)
Grade 3	0	0	1	0	0	0	0	0	0	0	0	0	1(2.2%)
**Infections and infestations**	1	0	0	0	0	1	0	0	0	0	0	0	2 (4.3%)
**Bronchitis**	**1**	**0**	**0**	**0**	**0**	**0**	**0**	**0**	**0**	**0**	**0**	**0**	**1 (2.2%)**
Grade 2	1	0	0	0	0	0	0	0	0	0	0	0	1 (2.2%)
**Sepsis**	**0**	**0**	**0**	**0**	**0**	**1**	**0**	**0**	**0**	**0**	**0**	**0**	**1 (2.2%)**
Grade 3	0	0	0	0	0	1	0	0	0	0	0	0	1 (2.2%)
**Injury, poisoning and procedural complications (not including IRR)**	0	0	0	2	0	0	0	0	0	0	0	0	2 (4.3%)
**Clavicle fracture**	**0**	**0**	**0**	**1**	**0**	**0**	**0**	**0**	**0**	**0**	**0**	**0**	**1 (2.2%)**
Grade 2	0	0	0	1	0	0	0	0	0	0	0	0	1 (2.2%)
**Fall**	**0**	**0**	**0**	**1**	**0**	**0**	**0**	**0**	**0**	**0**	**0**	**0**	**1 (2.2%)**
Grade 1	0	0	0	1	0	0	0	0	0	0	0	0	1 (2.2%)
**Investigations**	0	0	0	4	0	0	0	0	0	1	0	0	5 (10.9%)
**Blood alkaline phosphatase increased**	**0**	**0**	**0**	**1**	**0**	**0**	**0**	**0**	**0**	**0**	**0**	**0**	**1 (2.2%)**
Grade 1	0	0	0	1	0	0	0	0	0	0	0	0	1 (2.2%)
**Blood creatinine increased**	**0**	**0**	**0**	**0**	**0**	**0**	**0**	**0**	**0**	**1**	**0**	**0**	**1 (2.2%)**
Grade 1	0	0	0	0	0	0	0	0	0	1	0	0	1 (2.2%)
**Lymphocyte count decreased**	**0**	**0**	**0**	**1**	**0**	**0**	**0**	**0**	**0**	**0**	**0**	**0**	**1 (2.2%)**
Grade 2	0	0	0	1	0	0	0	0	0	0	0	0	1 (2.2%)
**Platelet count decreased**	**0**	**0**	**0**	**1**	**0**	**0**	**0**	**0**	**0**	**0**	**0**	**0**	**1 (2.2%)**
Grade 4	0	0	0	1	0	0	0	0	0	0	0	0	1 (2.2%)
**Urine output decreased**	**0**	**0**	**0**	**1**	**0**	**0**	**0**	**0**	**0**	**0**	**0**	**0**	**1 (2.2%)**
Grade 2	0	0	0	1	0	0	0	0	0	0	0	0	1 (2.2%)
**Metabolism and nutrition disorders**	0	0	0	4	0	0	0	1	2	0	0	0	7 (15.2%)
**Fluid overload**	**0**	**0**	**0**	**0**	**0**	**0**	**0**	**0**	**1**	**0**	**0**	**0**	**1 (2.2%)**
Grade 3	0	0	0	0	0	0	0	0	1	0	0	0	1 (2.2%)

MedDRA SOC<br>MedDRA PT	Cohort 1<br>(N=4)	Cohort 2<br>(N=3)	Cohort 3<br>(N=3)	Cohort 4<br>(N=6)	Cohort 5<br>(N=3)	Cohort 6A<br>(N=6)	Cohort 6B<br>(N=3)	Cohort 7<br>(N=4)	Cohort 8<br>(N=6)	Cohort 9<br>(N=3)	Cohort 10<br>(N=4)	Other<br>(N=1)	Total<br>N = 46<br>n (%)
**Hyperglycemia**	**0**	**0**	**0**	**1**	**0**	**0**	**0**	**0**	**0**	**0**	**0**	**0**	**1 (2.2%)**
Grade 3	0	0	0	1	0	0	0	0	0	0	0	0	1 (2.2%)
**Hypertriglyceridemia**	**0**	**0**	**0**	**0**	**0**	**0**	**0**	**0**	**1**	**0**	**0**	**0**	**1 (2.2%)**
Grade 2	0	0	0	0	0	0	0	0	1	0	0	0	1 (2.2%)
**Hypocalcemia**	**0**	**0**	**0**	**0**	**0**	**0**	**0**	**1**	**0**	**0**	**0**	**0**	**1 (2.2%)**
Grade 1	0	0	0	0	0	0	0	1	0	0	0	0	1 (2.2%)
**Hypomagnesaemia**	**0**	**0**	**0**	**1**	**0**	**0**	**0**	**0**	**0**	**0**	**0**	**0**	**1 (2.2%)**
Grade 2	0	0	0	1	0	0	0	0	0	0	0	0	1 (2.2%)
**Hyponatremia**	**0**	**0**	**0**	**1**	**0**	**0**	**0**	**0**	**0**	**0**	**0**	**0**	**1 (2.2%)**
Grade 1	0	0	0	1	0	0	0	0	0	0	0	0	1 (2.2%)
**Tumor lysis syndrome**	**0**	**0**	**0**	**1**	**0**	**0**	**0**	**0**	**0**	**0**	**0**	**0**	**1 (2.2%)**
Grade 3	0	0	0	1	0	0	0	0	0	0	0	0	1 (2.2%)
**Musculoskeletal and connective tissue disorders**	0	0	0	0	0	1	0	0	0	0	0	0	1 (2.2%)
**Limb discomfort**	**0**	**0**	**0**	**0**	**0**	**1**	**0**	**0**	**0**	**0**	**0**	**0**	**1 (2.2%)**
Grade 1	0	0	0	0	0	1	0	0	0	0	0	0	1 (2.2%)
**Nervous system disorders**	0	0	0	1	0	2	0	0	0	0	0	1	5 (10.9%)
**Dizziness**	**0**	**0**	**0**	**0**	**0**	**1**	**0**	**0**	**0**	**0**	**0**	**0**	**1 (2.2%)**
Grade 1	0	0	0	0	0	1	0	0	0	0	0	0	1 (2.2%)
**Headache**	**0**	**0**	**0**	**1**	**0**	**0**	**0**	**0**	**0**	**0**	**0**	**0**	**1 (2.2%)**
Grade 1	0	0	0	1	0	0	0	0	0	0	0	0	1 (2.2%)
**Lethargy**	**0**	**0**	**0**	**0**	**0**	**1**	**0**	**0**	**0**	**0**	**0**	**0**	**1 (2.2%)**
Grade 1	0	0	0	0	0	1	0	0	0	0	0	0	1(2.2%)
**Tremor**	**0**	**0**	**0**	**1**	**0**	**0**	**0**	**0**	**0**	**0**	**0**	**0**	**1(2.2%)**
Grade 1	0	0	0	1	0	0	0	0	0	0	0	0	1(2.2%)
**Memory loss**	**0**	**0**	**0**	**0**	**0**	**0**	**0**	**0**	**0**	**0**	**0**	**1**	**1(2.2%)**
Grade 1	0	0	0	0	0	0	0	0	0	0	0	1	1(2.2%)
**Neurotoxicity**	**0**	**0**	**0**	**0**	**0**	**0**	**0**	**0**	**0**	**0**	**0**	**1**	**1 (2.2%)**

MedDRA SOC<br>MedDRA PT	Cohort 1<br>(N=4)	Cohort 2<br>(N=3)	Cohort 3<br>(N=3)	Cohort 4<br>(N=6)	Cohort 5<br>(N=3)	Cohort 6A<br>(N=6)	Cohort 6B<br>(N=3)	Cohort 7<br>(N=4)	Cohort 8<br>(N=6)	Cohort 9<br>(N=3)	Cohort 10<br>(N=4)	Other<br>(N=1)	Total<br>N = 46<br>n (%)
Grade 1	0	0	0	0	0	0	0	0	0	0	0	1	1 (2.2%)
**Psychiatric disorders**	0	0	0	1	0	0	1	1	0	0	0	1	4 (8.7%)
**Confusional state**	**0**	**0**	**0**	**1**	**0**	**0**	**0**	**1**	**0**	**0**	**0**	**1**	**3 (6.5%)**
Grade 1	0	0	0	1	0	0	0	0	0	0	0	1	2 (4.3%)
Grade 3	0	0	0	0	0	0	0	1	0	0	0	0	1(2.2%)
**Insomnia**	**0**	**0**	**0**	**0**	**0**	**0**	**1**	**0**	**0**	**0**	**0**	**0**	**1 (2.2%)**
Grade 1	0	0	0	0	0	0	1	0	0	0	0	0	1 (2.2%)
**Renal and urinary disorders**	0	0	0	1	0	1	0	0	0	0	0	0	2 (4.3%)
**Acute kidney injury**	**0**	**0**	**0**	**1**	**0**	**0**	**0**	**0**	**0**	**0**	**0**	**0**	**1 (2.2%)**
Grade 5	0	0	0	1	0	0	0	0	0	0	0	0	1 (2.2%)
**Dysuria**	**0**	**0**	**0**	**0**	**0**	**1**	**0**	**0**	**0**	**0**	**0**	**0**	**1 (2.2%)**
Grade 2	0	0	0	0	0	1	0	0	0	0	0	0	1 (2.2%)
**Respiratory, thoracic, and mediastinal disorders**	1	0	0	1	0	4	0	0	0	0	0	0	6(13.0%)
**Dyspnea**	**1**	**0**	**0**	**0**	**0**	**1**	**0**	**0**	**0**	**0**	**0**	**0**	**2 (4.3%)**
Grade 2	0	0	0	0	0	1	0	0	0	0	0	0	1 (2.2%)
Grade 3	1	0	0	0	0	0	0	0	0	0	0	0	1 (2.2%)
**Hypoxia**	**0**	**0**	**0**	**0**	**0**	**1**	**0**	**0**	**0**	**0**	**0**	**0**	**1 (2.2%)**
Grade 3	0	0	0	0	0	1	0	0	0	0	0	0	1 (2.2%)
**Acute hypoxemic resp. failure**	0	0	0	1	0	0	0	0	0	0	0	0	1 (2.2%)
Grade 4	0	0	0	1	0	0	0	0	0	0	0	0	1 (2.2%)
**Pulmonary infiltrates**	**0**	**0**	**0**	**0**	**0**	**1**	**0**	**0**	**0**	**0**	**0**	**0**	**1 (2.2%)**
Grade 3	0	0	0	0	0	1	0	0	0	0	0	0	1 (2.2%)
**Pulmonary oedema**	**0**	**0**	**0**	**1**	**0**	**0**	**0**	**0**	**0**	**0**	**0**	**0**	**1 (2.2%)**
Grade 2	0	0	0	1	0	0	0	0	0	0	0	0	1 (2.2%)
**Tachypnea**	**0**	**0**	**0**	**0**	**0**	**1**	**0**	**0**	**0**	**0**	**0**	**0**	**1 (2.2%)**
Grade 1	0	0	0	0	0	1	0	0	0	0	0	0	1 (2.2%)
**Pleural Effusion**	0	0	0	1	0	0	0	0	0	0	0	0	1 (2.2%)

MedDRA SOC<br>MedDRA PT	Cohort 1<br>(N=4)	Cohort 2<br>(N=3)	Cohort 3<br>(N=3)	Cohort 4<br>(N=6)	Cohort 5<br>(N=3)	Cohort 6A<br>(N=6)	Cohort 6B<br>(N=3)	Cohort 7<br>(N=4)	Cohort 8<br>(N=6)	Cohort 9<br>(N=3)	Cohort 10<br>(N=4)	Other<br>(N=1)	Total<br>N = 46<br>n (%)
Grade 3	0	0	0	1	0	0	0	0	0	0	0	0	1 (2.2%)
**Skin and subcutaneous tissue disorders**	0	0	0	0	0	1	0	0	0	0	0	0	1 (2.2%)
**Erythema**	**0**	**0**	**0**	**0**	**0**	**1**	**0**	**0**	**0**	**0**	**0**	**0**	**1 (2.2%)**
Grade 1	0	0	0	0	0	1	0	0	0	0	0	0	1 (2.2%)
**Vascular disorders**	3	0	0	1	0	1	0	0	0	1	0	0	6(13.0%)
**Flushing**	**1**	**0**	**0**	**0**	**0**	**1**	**0**	**0**	**0**	**0**	**0**	**0**	**2 (4.3%)**
Grade 1	0	0	0	0	0	1	0	0	0	0	0	0	1 (2.2%)
Grade 2	1	0	0	0	0	0	0	0	0	0	0	0	1 (2.2%)
**Hypotension**	**2**	**0**	**0**	**1**	**0**	**0**	**0**	**0**	**0**	**1**	**0**	**0**	**4 (8.7%)**
Grade 1	0	0	0	0	0	0	0	0	0	1	0	0	1 (2.2%)
Grade 2	1	0	0	1	0	0	0	0	0	0	0	0	2 (4.3%)
Grade 3	1	0	0	1	0	0	0	0	0	0	0	0	1 (2.2%)
**Hypertension**	**0**	**0**	**0**	**0**	**0**	**0**	**0**	**0**	**0**	**1**	**0**	**0**	**1(2.2%)**
Grade 1	0	0	0	0	0	0	0	0	0	1	0	0	1(2.2%)
**Shock**	0	0	0	1	0	0	0	0	0	0	0	0	1 (2.2%)
Grade 4	0	0	0	1	0	0	0	0	0	0	0	0	1 (2.2%)

Table S5: Incidence of adverse events by MedDRA PT and worst CTCAE Grade ≥3 severity occurring in patients treated with APVO436 in Study 5001 regardless of any relationship with the study drug APVO436

MedDRA SOC/PT	Cohorts											Total
	Cohort 1 (N=4)	Cohort 2 (N=3)	Cohort 3 (N=3)	Cohort 4 (N=6)	Cohort 5 (N=3)	Cohort 6A (N=6)	Cohort 6B (N=3)	Cohort 7 (N=4)	Cohort 8 (N=6)	Cohort 9 (N=3)	Cohort 10 (N=4)	N = 46 n (%)
**Blood and lymphatic system disorders**												
**Anemia**	**1**	**1**	**0**	**3**	**0**	**0**	**0**	**1**	**0**	**0**	**1**	**7 (15.2%)**
Grade 3	1	1	0	3	0	0	0	1	0	0	1	7 (15.2%)
**Febrile neutropenia**	**1**	**1**	**0**	**1**	**2**	**2**	**1**	**0**	**2**	**0**	**0**	**10 (21.7%)**
Grade 3	1	1	0	1	2	2	1	0	2	0	0	10 (21.7%)
**Leukocytosis**	**1**	**0**	**0**	**0**	**0**	**0**	**0**	**0**	**0**	**0**	**0**	**2 (4.3%)**
Grade 3	1	0	0	0	0	0	0	0	0	0	0	2 (4.3%)
**Thrombocytopenia**	**0**	**0**	**0**	**0**	**0**	**0**	**0**	**1**	**1**	**0**	**0**	**2 (4.3%)**
Grade 4	0	0	0	0	0	0	0	1	1	0	0	2 (4.3%)
**Cardiac disorders**												
**Acute myocardial infarction**	**0**	**0**	**0**	**0**	**0**	**0**	**0**	**0**	**1**	**0**	**0**	**1 (2.2%)**
Grade 3	0	0	0	0	0	0	0	0	1	0	0	1 (2.2%)
**Atrial fibrillation**	**0**	**0**	**0**	**0**	**0**	**1**	**0**	**0**	**0**	**0**	**0**	**1 (2.2%)**
Grade 3	0	0	0	0	0	1	0	0	0	0	0	1 (2.2%)
**Supraventricular tachycardia**	**0**	**0**	**0**	**0**	**0**	**1**	**0**	**0**	**0**	**0**	**0**	**1 (2.2%)**
Grade 3	0	0	0	0	0	1	0	0	0	0	0	1 (2.2%)
**Gastrointestinal disorders**												
**Abdominal pain**	**0**	**0**	**1**	**0**	**0**	**0**	**0**	**0**	**0**	**0**	**0**	**1 (2.2%)**
Grade 3	0	0	1	0	0	0	0	0	0	0	0	1 (2.2%)
**Diarrhea**	**0**	**0**	**1**	**0**	**0**	**1**	**0**	**0**	**0**	**0**	**0**	**2 (4.3%)**
Grade 3	0	0	1	0	0	1	0	0	0	0	0	2 (4.3%)
**Lower gastrointestinal hemorrhage**	**0**	**0**	**0**	**0**	**0**	**1**	**0**	**0**	**0**	**0**	**0**	**1 (2.2%)**
Grade 3	0	0	0	0	0	1	0	0	0	0	0	1 (2.2%)
**Nausea**	**0**	**0**	**0**	**1**	**0**	**0**	**0**	**0**	**0**	**0**	**0**	**1 (2.2%)**
Grade 3	0	0	0	1	0	0	0	0	0	0	0	1 (2.2%)
**Oral pain**	**0**	**0**	**0**	**0**	**0**	**0**	**0**	**0**	**0**	**0**	**1**	**1 (2.2%)**
Grade 3	0	0	0	0	0	0	0	0	0	0	1	1 (2.2%)
**Vomiting**	**0**	**0**	**0**	**0**	**0**	**1**	**0**	**0**	**0**	**0**	**0**	**1 (2.2%)**
Grade 3	0	0	0	0	0	1	0	0	0	0	0	1 (2.2%)
**General disorders and administration site conditions**												
**Asthenia**	**0**	**0**	**0**	**0**	**0**	**1**	**0**	**0**	**0**	**1**	**0**	**2 (4.3%)**
Grade 3	0	0	0	0	0	1	0	0	0	1	0	2 (4.3%)
**Chills**	1	0	0	0	0	0	0	0	0	0	0	1 (2.2%)
Grade 3	1	0	0	0	0	0	0	0	0	0	0	1 (2.2%)
**Fatigue**	**0**	**0**	**0**	**1**	**0**	**0**	**0**	**0**	**0**	**0**	**0**	**1 (2.2%)**
Grade 3	0	0	0	1	0	0	0	0	0	0	0	1 (2.2%)
**Generalized edema**	**0**	**0**	**0**	**1**	**0**	**0**	**0**	**0**	**0**	**0**	**1**	**2 (4.3%)**
Grade 3	0	0	0	1	0	0	0	0	0	0	1	2 (4.3%)
**Pyrexia**	**0**	**0**	**0**	**1**	**0**	**0**	**0**	**1**	**0**	**1**	**0**	**3 (6.5%)**

MedDRA SOC/PT	Cohorts											Total
	Cohort 1 (N=4)	Cohort 2 (N=3)	Cohort 3 (N=3)	Cohort 4 (N=6)	Cohort 5 (N=3)	Cohort 6A (N=6)	Cohort 6B (N=3)	Cohort 7 (N=4)	Cohort 8 (N=6)	Cohort 9 (N=3)	Cohort 10 (N=4)	N = 46 n (%)
Grade 3	0	0	0	1	0	0	0	1	0	1	0	3 (6.5%)
**Disease progression**	**0**	**0**	**0**	**0**	**0**	**0**	**0**	**0**	**1**	**0**	**0**	**1 (2.2%)**
Grade 5	0	0	0	0	0	0	0	0	1	0	0	1 (2.2%)
**Immune system disorders**												
**Cytokine release syndrome**	**1**	**0**	**0**	**1**	**0**	**1**	**0**	**0**	**1**	**0**	**0**	**4 (8.7%)**
Grade 3	1	0	0	0	0	1	0	0	1	0	0	3 (6.5%)
Grade 4	0	0	0	1	0	0	0	0	0	0	0	1 (2.2%)
**Infections and infestations**												
**Anal abscess**	**0**	**0**	**0**	**0**	**1**	**0**	**0**	**0**	**0**	**0**	**0**	**1 (2.2%)**
Grade 3	0	0	0	0	1	0	0	0	0	0	0	1 (2.2%)
**Cellulitis**	**0**	**0**	**0**	**1**	**0**	**2**	**0**	**0**	**0**	**0**	**0**	**3 (6.5%)**
Grade 3	0	0	0	1	0	1	0	0	0	0	0	2 (4.3%)
Grade 5	0	0	0	0	0	1	0	0	0	0	0	1 (2.2%)
**Enteritis infectious**	**0**	**0**	**0**	**1**	**0**	**0**	**0**	**0**	**0**	**0**	**0**	**1 (2.2%)**
Grade 3	0	0	0	1	0	0	0	0	0	0	0	1 (2.2%)
**Localized infection**	**0**	**0**	**0**	**0**	**0**	**0**	**0**	**0**	**1**	**0**	**0**	**1 (2.2%)**
Grade 3	0	0	0	0	0	0	0	0	1	0	0	1 (2.2%)
**Pneumonia**	**0**	**0**	**0**	**0**	**0**	**1**	**0**	**0**	**0**	**0**	**0**	**1 (2.2%)**
Grade 5	0	0	0	0	0	1	0	0	0	0	0	1 (2.2%)
**Sepsis**	**1**	**0**	**1**	**0**	**0**	**2**	**0**	**0**	**1**	**0**	**0**	**5 (10.9%)**
Grade 3	0	0	0	0	0	1	0	0	1	0	0	2 (4.3%)
Grade 4	1	0	0	0	0	0	0	0	0	0	0	1 (2.2%)
Grade 5	0	0	1	0	0	1	0	0	0	0	0	2 (4.3%)
**Septic shock**	**0**	**0**	**0**	**0**	**0**	**1**	**0**	**0**	**0**	**0**	**0**	**1 (2.2%)**
Grade 4	0	0	0	0	0	1	0	0	0	0	0	1 (2.2%)
**Sinusitis**	**0**	**0**	**0**	**0**	**0**	**0**	**0**	**0**	**0**	**0**	**1**	**1 (2.2%)**
Grade 3	0	0	0	0	0	0	0	0	0	0	1	1 (2.2%)
**Tooth infection**	**0**	**0**	**0**	**0**	**0**	**0**	**0**	**0**	**0**	**0**	**1**	**1 (2.2%)**
Grade 3	0	0	0	0	0	0	0	0	0	0	1	1 (2.2%)
**Injury, poisoning and procedural complications**												
**Infusion related reaction**	**1**	**0**	**1**	**0**	**0**	**0**	**0**	**0**	**0**	**0**	**0**	**2 (4.3%)**
Grade 3	1	0	1	0	0	0	0	0	0	0	0	2 (4.3%)
**Investigations**												
**Alanine aminotransferase increased**	**0**	**0**	**0**	**1**	**0**	**0**	**0**	**0**	**0**	**0**	**0**	**1 (2.2%)**
Grade 3	0	0	0	1	0	0	0	0	0	0	0	1 (2.2%)
**Aspartate aminotransferase increased**	**0**	**0**	**0**	**1**	**0**	**0**	**0**	**0**	**1**	**0**	**0**	**2 (4.3%)**
Grade 3	0	0	0	1	0	0	0	0	0	0	0	1 (2.2%)
Grade 4	0	0	0	0	0	0	0	0	1	0	0	1 (2.2%)
**Blood alkaline phosphatase increased**	**0**	**0**	**0**	**0**	**0**	**0**	**0**	**0**	**1**	**0**	**0**	**1 (2.2%)**
Grade 3	0	0	0	0	0	0	0	0	1	0	0	1 (2.2%)
**Lymphocyte count decreased**	**0**	**0**	**0**	**0**	**0**	**0**	**0**	**1**	**1**	**0**	**1**	**3 (6.5%)**
Grade 3	0	0	0	0	0	0	0	0	1	0	0	1 (2.2%)

MedDRA SOC/PT	Cohorts											Total
	Cohort 1 (N=4)	Cohort 2 (N=3)	Cohort 3 (N=3)	Cohort 4 (N=6)	Cohort 5 (N=3)	Cohort 6A (N=6)	Cohort 6B (N=3)	Cohort 7 (N=4)	Cohort 8 (N=6)	Cohort 9 (N=3)	Cohort 10 (N=4)	N = 46 n (%)
Grade 4	0	0	0	0	0	0	0	1	0	0	1	2 (4.3%)
**Neutrophil count decreased**	**0**	**0**	**0**	**0**	**0**	**1**	**0**	**0**	**0**	**0**	**0**	**1 (2.2%)**
Grade 4	0	0	0	0	0	1	0	0	0	0	0	1 (2.2%)
**Platelet count decreased**	**0**	**1**	**0**	**2**	**0**	**0**	**0**	**0**	**2**	**0**	**0**	**5 (10.9%)**
Grade 4	0	1	0	2	0	0	0	0	2	0	0	5 (10.9%)
**White blood cell count decreased**	**0**	**0**	**0**	**1**	**0**	**1**	**0**	**0**	**1**	**0**	**0**	**3 (6.5%)**
Grade 3	0	0	0	0	0	0	0	0	1	0	0	1 (2.2%)
Grade 4	0	0	0	1	0	1	0	0	0	0	0	2 (4.3%)
**Metabolism and nutrition disorders**												
**Fluid overload**	**0**	**0**	**0**	**0**	**0**	**0**	**0**	**0**	**1**	**0**	**0**	**1 (2.2%)**
Grade 3	0	0	0	0	0	0	0	0	1	0	0	1 (2.2%)
**Hyperglycemia**	**1**	**0**	**1**	**2**	**0**	**0**	**0**	**2**	**0**	**0**	**0**	**6 (13.0%)**
Grade 3	1	0	1	2	0	0	0	2	0	0	0	6 (13.0%)
**Hypermagnesemia**	**0**	**0**	**0**	**1**	**0**	**0**	**0**	**0**	**0**	**0**	**0**	**1 (2.2%)**
Grade 3	0	0	0	1	0	0	0	0	0	0	0	1 (2.2%)
**Hypoalbuminemia**	**0**	**0**	**0**	**1**	**0**	**0**	**0**	**0**	**0**	**0**	**0**	**1 (2.2%)**
Grade 3	0	0	0	1	0	0	0	0	0	0	0	1 (2.2%)
**Hyponatremia**	**0**	**0**	**0**	**0**	**0**	**1**	**0**	**0**	**0**	**0**	**0**	**1 (2.2%)**
Grade 3	0	0	0	0	0	1	0	0	0	0	0	1 (2.2%)
**Lactic acidosis**	**0**	**0**	**0**	**0**	**0**	**0**	**1**	**0**	**0**	**0**	**0**	**1 (2.2%)**
Grade 3	0	0	0	0	0	0	1	0	0	0	0	1 (2.2%)
**Tumor lysis syndrome**	**0**	**0**	**0**	**1**	**0**	**0**	**0**	**0**	**1**	**0**	**0**	**2 (4.3%)**
Grade 3	0	0	0	1	0	0	0	0	0	0	0	1 (2.2%)
Grade 5	0	0	0	0	0	0	0	0	1	0	0	1 (2.2%)
**Musculoskeletal and connective tissue disorders**												
**Arthralgia**	**0**	**0**	**0**	**0**	**0**	**0**	**0**	**0**	**0**	**0**	**1**	**1 (2.2%)**
Grade 3	0	0	0	0	0	0	0	0	0	0	1	1 (2.2%)
**Nervous system disorders**												
**Encephalopathy**	**0**	**0**	**0**	**0**	**0**	**1**	**0**	**0**	**0**	**0**	**0**	**1 (2.2%)**
Grade 3	0	0	0	0	0	1	0	0	0	0	0	1 (2.2%)
**Psychiatric disorders**												
**Confusional state**	**0**	**0**	**0**	**0**	**0**	**0**	**0**	**1**	**0**	**0**	**1**	**2 (4.3%)**
Grade 3	0	0	0	0	0	0	0	1	0	0	1	2 (4.3%)
**Renal and urinary disorders**												
**Acute kidney injury**	**1**	**0**	**0**	**2**	**0**	**0**	**0**	**0**	**0**	**0**	**0**	**3 (6.5%)**
Grade 3	1	0	0	1	0	0	0	0	0	0	0	2 (4.3%)
Grade 5	0	0	0	1	0	0	0	0	0	0	0	1 (2.2%)
**Respiratory, thoracic and mediastinal disorders**												
**Acute respiratory failure**	**0**	**0**	**0**	**1**	**0**	**1**	**0**	**0**	**0**	**0**	**0**	**2 (4.3%)**
Grade 3	0	0	0	0	0	1	0	0	0	0	0	1 (2.2%)
Grade 4	0	0	0	1	0	0	0	0	0	0	0	1 (2.2%)

MedDRA SOC/PT	Cohorts											Total
	**Cohort 1 (N=4)**	**Cohort 2 (N=3)**	**Cohort 3 (N=3)**	**Cohort 4 (N=6)**	**Cohort 5 (N=3)**	**Cohort 6A (N=6)**	**Cohort 6B (N=3)**	**Cohort 7 (N=4)**	**Cohort 8 (N=6)**	**Cohort 9 (N=3)**	**Cohort 10 (N=4)**	**N = 46 n (%)**
**Hypoxia**	**0**	**0**	**0**	**1**	**0**	**1**	**1**	**0**	**0**	**0**	**0**	**3 (6.5%)**
Grade 3	0	0	0	1	0	1	1	0	0	0	0	3 (6.5%)
**Pleural effusion**	**0**	**0**	**0**	**1**	**0**	**0**	**0**	**0**	**0**	**0**	**0**	**1 (2.2%)**
Grade 3	0	0	0	1	0	0	0	0	0	0	0	1 (2.2%)
**Pulmonary edema**	**0**	**0**	**0**	**0**	**0**	**1**	**0**	**0**	**0**	**0**	**0**	**1 (2.2%)**
Grade 3	0	0	0	0	0	1	0	0	0	0	0	1 (2.2%)
**Respiratory failure**	**1**	**0**	**0**	**0**	**0**	**0**	**0**	**0**	**0**	**0**	**0**	**1 (2.2%)**
Grade 4	1	0	0	0	0	0	0	0	0	0	0	1 (2.2%)
**Skin and subcutaneous tissue disorders**												
**Rash**	**1**	**0**	**0**	**0**	**0**	**0**	**0**	**0**	**0**	**0**	**0**	**1 (2.2%)**
Grade 3	1	0	0	0	0	0	0	0	0	0	0	1 (2.2%)
**Vascular disorders**												
**Deep vein thrombosis**	**0**	**0**	**0**	**0**	**0**	**1**	**0**	**0**	**0**	**0**	**0**	**1 (2.2%)**
Grade 3	0	0	0	0	0	1	0	0	0	0	0	1 (2.2%)
**Hypertension**	**0**	**1**	**1**	**0**	**0**	**0**	**0**	**0**	**0**	**0**	**0**	**2 (4.3%)**
Grade 3	0	1	1	0	0	0	0	0	0	0	0	2 (4.3%)
**Hypotension**	**0**	**0**	**0**	**1**	**0**	**0**	**1**	**0**	**0**	**0**	**0**	**2 (4.3%)**
Grade 3	0	0	0	1	0	0	1	0	0	0	0	2 (4.3%)
**Shock**	**0**	**0**	**0**	**1**	**0**	**0**	**0**	**0**	**0**	**0**	**0**	**1 (2.2%)**
Grade 4	0	0	0	1	0	0	0	0	0	0	0	1 (2.2%)

Note: When the same event was reported twice for the same patient, it was only counted once, and the highest grade (worst grade) was captured.

Table S6. Listing of all APVO436-related Grade ≥3 AE occurring in patients treated with APVO436 in Study 5001

UPN	Cohort#	AE Reported Term (CTCAE Grade)	Start Date (CxDx); days from IC	End Date (days from IC)	Total Duration (days)	Outcome	Changes to Drug Dose or Schedule	DLT (Y/N)
UPN02	1	CRS (3)	C6D1; 148	155	8	Resolved	None	No
		Rigors (3)	C6D1; 148	150	3	Resolved	None	No
		Chills (3)	C6D1; 148	150	3	Resolved	None	No
		Dyspnea (3)	C6D1; 148	150	3	Resolved	None	No
		Hypotension (3)	C6D1; 148	150	3	Resolved	None	No
UPN04	1	IRR – Fever (3)	C1D8; 14	16	3	Resolved	None	No
UPN10	3	IRR (3)	C2D1; 35	36	2	Resolved	None	No
UPN12	4	Anemia (3)	C2D1; 47	NA	NA	NR	None	No
		Nausea (3)	C1D25; 40	NA	NA	NR	None	No
		Acute kidney failure (5)	C2D1; 55	55	1	Fatal	DD	No
UPN13	4	Tumor lysis syndrome (3)	C1D2; 14	15	2	Resolved	None	No
		Tumor lysis syndrome (3)	C4D1; 98	99	2	Resolved	None	No
		Platelet count decreased (3)	C4D15; 117	123	7	Resolved	None	No
		Platelet count decreased (4)	C4D22; 124	173	50	Resolved	None	No
		Anemia (3)	C5D1; 131	138	8	Resolved	None	No
		Hyperglycemia (3)	C5D15; 145	146	2	Resolved	None	No
		Anemia (3)	C7D22; 204	211	8	Resolved	None	No
		Platelet count decreased (3)	C6D15; 174	175	2	Resolved	None	No
		Platelet count decreased (4)	C6D22*; 176	208	33	Resolved	None	No
		Anemia (3)	C6D22; 176	187	12	Resolved	None	No
		Anemia (3)	C7D8; 190	193	4	Resolved	None	No

UPN	Cohort#	AE Reported Term (CTCAE Grade)	Start Date (CxDx); days from IC	End Date (days from IC)	Total Duration (days)	Outcome	Changes to Drug Dose or Schedule	DLT (Y/N)
UPN16	4	Fatigue (3)	C1D5; 19	NA	NA	NR	None	No
		CRS (4)	C1D5; 19	21	NA	Resolved	DPD	No
		Intermittent Hypoxia (3)	C1D2; 14	NA	NA	NR	TI	No
		Generalized Edema (3)	C1D3; 16	19	4	Resolved	None	No
		Pleural Effusion (3)	C1D5; 18	24	7	Resolved	None	No
		Respiratory Failure – Acute (4)	C1D5; 19	21	3	Resolved	None	Yes
		Acute Kidney Injury (3)	C1D5; 19	22	4	Resolved	None	Yes
		AST Increased (3)	C1D8; 20	24	5	Resolved	None	No
		ALT Increased (3)	C1D8; 20	NA	NA	NR	None	No
		Undifferentiated Shock (4)	C1D5; 17	21	5	Resolved	DPD	Yes
UPN20	6A	Sepsis (3)	C6D15; 165	169	5	Resolved	TI	No
		Diarrhea (3)	C6D15; 165	169	5	Resolved	TI	No
		Vomiting (3)	C6D15; 165	169	5	Resolved	TI	No
UPN22	6A	CRS (3)	C1D3; 6	15	10	Resolved	DD	No
		Hypoxia (3)	C1D3; 6	10	5	Resolved	DD	No
		Pulmonary oedema (3)	C1D3; 6	13	8	Resolved	DD	No
		Pulmonary infiltrates (3)	C1D3; 6	15	10	Resolved	DD	No
UPN24	6A	Generalized weakness (3)	C1D5; 11	38	28	Resolved	DD	No
UPN31	7	Confusion (3)	C5D1; 120	120	1	Resolved	None	No
UPN38	8	N-STEMI Secondary to CRS (3)	C1D1; 5	6	2	Resolved	TI	No
		Fluid overload (3)	C1D11; 21	23	3	Resolved	None	No
		Hypotension (3)	C1D1; 5	5	1	Resolved	TI	No
		CRS (3)	C1D1; 5	6	2	Resolved	TI	No

Multiple events for the same term and patient have been reported as 1 event only unless same event was reported for 2 different Grades i.e., worsened
NA: not applicable; IC: Informed consent;

UPN	Cohort#	AE Reported Term (CTCAE Grade)	Start Date (CxDx); days from IC	End Date (days from IC)	Total Duration (days)	Outcome	Changes to Drug Dose or Schedule	DLT (Y/N)

NR: not resolved; TI: temporarily interrupted (including interruption of infusion or discontinuation of infusion for the day of the AE); DPD: drug permanently discontinued; DD: next dose delayed; CRS: cytokine release syndrome; IC: informed consent; DLT: dose limiting toxicity; C: Cycle; D: Day;

UPN12: This patient had a Grade 2 CRS and subsequently complicated by acute kidney failure that was fatal. See the SAE listings in Table 3.

Table S7. Febrile neutropenia (FBN) in AML/MDS patients treated with APVO436 in Study 5001

Subject	Cohort	Preferred Term	Start Date	End Date	Outcome	Severity (CTCAE Grade)	Relationship to Study Treatment?	Changes to Drug Dose or Schedule	Is the Adverse Event serious?
UPN03	1	FBN	D21	D46	Resolved	Grade 3	Not related	None	Yes
UPN07	2	FBN	D50	NA	Not resolved	Grade 3	Not Related	None	No
UPN14	4	FBN	D39	D42	Resolved	Grade 3	Not Related	None	No
UPN14	4	FBN	D14	D19	Resolved	Grade 3	Not Related	None	No
UPN14	4	FBN	D31	D32	Resolved	Grade 3	Not Related	None	No
UPN16	4	FBN	D14	D14	Resolved	Grade 3	Not Related	None	No
UPN17	5	FBN	D65	D67	Resolved	Grade 3	Not Related	None	No
UPN18	5	FBN	D26	NA	Resolving	Grade 3	Not Related	None	No
UPN23	6A	FBN	D84	D86	Resolved	Grade 3	Not Related	None	No
UPN24	6A	FBN	D31	D32	Resolved	Grade 3	Not Related	None	No
UPN26	6B	FBN	D24	D25	Resolved	Grade 3	Not Related	None	No
UPN36	8	FBN	D42	D48	Resolved	Grade 3	Not Related	DD	Yes

FBN: febrile neutropenia; NA: not applicable; DD: next dose delayed; D: Day from informed consent

FBN is defined as Grade 3 or Grade 4 neutropenia with ANC<1,000/μL accompanied by fever with a single temperature >38.3°C (101°F) or sustained elevated temperatures >38°C (100.4°F) for more than hour.

Table S8: Incidence of all APVO436-related SAE by MedDRA PT and worst CTCAE Grade occurring in patients treated with APVO436 in Study

MedDRA SOC<br>MedDRA PT	Cohorts												Total
	Cohort 1	Cohort 2	Cohort 3	Cohort 4	Cohort 5	Cohort 6A	Cohort 6B	Cohort 7	Cohort 8	Cohort 9	Cohort 10	Other	N = 46
	(N=4)	(N=3)	(N=3)	(N=6)	(N=3)	(N=6)	(N=3)	(N=4)	(N=6)	(N=3)	(N=4)	(N=1)	n (%)
**Cardiac disorders**													
**Acute myocardial infarction**	**0**	**0**	**0**	**0**	**0**	**0**	**0**	**0**	**1**	**0**	**0**	**0**	**1 (2.2%)**
Grade 3	0	0	0	0	0	0	0	0	1	0	0	0	1 (2.2%)
**General disorders and administration site conditions**													
**Asthenia**	**0**	**0**	**0**	**0**	**0**	**1**	**0**	**0**	**0**	**0**	**0**	**0**	**1 (2.2%)**
Grade 3	0	0	0	0	0	1	0	0	0	0	0	0	1 (2.2%)
**Chills**	**1**	**0**	**0**	**0**	**0**	**0**	**0**	**0**	**0**	**0**	**0**	**0**	**1 (2.2%)**
Grade 3	1	0	0	0	0	0	0	0	0	0	0	0	1 (2.2%)
**Rigors**	**1**	**0**	**0**	**0**	**0**	**0**	**0**	**1**	**1**	**0**	**0**	**0**	3(6.5%)
Grade 2	0	0	0	0	0	0	0	1	1	0	0	0	2(4.3%)
Grade 3	1	0	0	0	0	0	0	0	0	0	0	0	1(2.2%)
**Pyrexia**	**1**	**0**	**0**	**0**	**0**	**0**	**0**	**0**	**1**	**0**	**0**	**0**	**2(4.3%)**
Grade 2	0	0	0	0	0	0	0	0	1	0	0	0	1(2.2%)
Grade 3	1	0	0	0	0	0	0	0	0	0	0	0	1(2.2%)
**Immune system disorders**													
**Cytokine release syndrome**	**1**	**0**	**0**	**3**	**0**	**1**	**0**	**1**	**1**	**0**	**0**	**0**	**7 (15.2%)**
Grade 1	0	0	0	1	0	0	0	0	0	0	0	0	1 (2.2%)
Grade 2	0	0	0	1	0	0	0	1	0	0	0	0	2 (4.3%)
Grade 3	1	0	0	0	0	1	0	0	1	0	0	0	3 (6.5%)
Grade 4	0	0	0	1	0	0	0	0	0	0	0	0	1 (2.2%)
**Infections and infestations**													
**Sepsis**	**0**	**0**	**0**	**0**	**0**	**1**	**0**	**0**	**0**	**0**	**0**	**0**	**1 (2.2%)**
Grade 3	0	0	0	0	0	1	0	0	0	0	0	0	1 (2.2%)
**Injury, poisoning and procedural complications**													
**Infusion related reaction**	**1**	**0**	**0**	**0**	**1**	**0**	**0**	**1**	**0**	**0**	**0**	**0**	**3 (6.5%)**

MedDRA SOC<br>MedDRA PT	Cohort 1<br>(N=4)	Cohort 2<br>(N=3)	Cohort 3<br>(N=3)	Cohort 4<br>(N=6)	Cohort 5<br>(N=3)	Cohort 6A<br>(N=6)	Cohort 6B<br>(N=3)	Cohort 7<br>(N=4)	Cohort 8<br>(N=6)	Cohort 9<br>(N=3)	Cohort 10<br>(N=4)	Other<br>(N=1)	Total<br>N = 46<br>n (%)
Grade 1	1	0	0	0	1	0	0	0	0	0	0	0	2 (4.3%)
Grade 2	0	0	0	0	0	0	0	1	0	0	0	0	1 (2.2%)
**Metabolism and nutrition disorders**													
**Fluid overload**	**0**	**0**	**0**	**0**	**0**	**0**	**0**	**0**	**1**	**0**	**0**	**0**	**1 (2.2%)**
Grade 3	0	0	0	0	0	0	0	0	1	0	0	0	1 (2.2%)
**Nervous system disorders**													
**Neurotoxicity**	**0**	**0**	**0**	**0**	**0**	**0**	**0**	**0**	**0**	**0**	**0**	**1**	**1 (2.2%)**
Grade 1	0	0	0	0	0	0	0	0	0	0	0	1	1 (2.2%)
**Renal and urinary disorders**													
**Acute kidney injury**	**0**	**0**	**0**	**2**	**0**	**0**	**0**	**0**	**0**	**0**	**0**	**0**	**2(4.3%)**
Grade 3	**0**	**0**	**0**	**1**	**0**	**0**	**0**	**0**	**0**	**0**	**0**	**0**	**1(2.2%)**
Grade 5	0	0	0	1	0	0	0	0	0	0	0	0	1 (2.2%)
**Respiratory, thoracic, and mediastinal disorders**													
**Acute hypoxemic respiratory failure/ARDS**	**0**	**0**	**0**	**1**	**0**	**0**	**0**	**0**	**0**	**0**	**0**	**0**	**1 (2.2%)**
Grade 4	0	0	0	1	0	0	0	0	0	0	0	0	1 (2.2%)
**Dyspnea**	**1**	**0**	**0**	**0**	**0**	**1**	**0**	**0**	**0**	**0**	**0**	**0**	**2 (4.3%)**
Grade 2	0	0	0	0	0	1	0	0	0	0	0	0	1 (2.2%)
Grade 3	1	0	0	0	0	0	0	0	0	0	0	0	1 (2.2%)
**Hypoxia**	**0**	**0**	**0**	**0**	**0**	**1**	**0**	**0**	**0**	**0**	**0**	**0**	**1 (2.2%)**
Grade 3	0	0	0	0	0	1	0	0	0	0	0	0	1 (2.2%)
**Pulmonary infiltrates**	**0**	**0**	**0**	**0**	**0**	**1**	**0**	**0**	**0**	**0**	**0**	**0**	**1 (2.2%)**
Grade 3	0	0	0	0	0	1	0	0	0	0	0	0	1 (2.2%)
**Pleural effusion**													
Grade 4	0	0	0	1	0	0	0	0	0	0	0	0	1(2.2%)
**Vascular disorders**													
**Hypotension**	**1**	**0**	**0**	**0**	**0**	**0**	**0**	**0**	**1**	**0**	**0**	**0**	**2 (4.3%)**
Grade 3	1	0	0	0	0	0	0	0	1	0	0	0	**2 (4.3%)**

Table S9. Predictors of APVO436-Related SAE and Lack of SAE Impact on Disease Progression or Survival Time

	SAE<br>N=13	No SAE<br>N=33	P-Value
**Age**	Mean: 68.7 ±2.4<br>95% CI: 63.4, 74.0<br>Median: 69<br>Range: 46-78	Mean: 64.1±2.5<br>95% CI: 59.0, 69.3<br>Median: 69<br>Range: 18-82	0.3
**Gender**	M: 53.8%<br>F: 46.2%	M: 51.5%<br>F: 48.5%	0.9
**Race**	C: 76.9%<br>HL: 7.7%<br>W-HL: 7.7%<br>A: 7.7%<br>B: 0%	C: 69.7%<br>HL: 3%<br>W-HL: 15.2%<br>A: 3%<br>B: 9.1%	1.0
**Dose (μg/kg)**	Mean: 0.23±0.05<br>95% CI: 0.11, 0.35<br>Median: 0.20<br>Range: 0.004-0.62	Mean: 0.31±0.047<br>95% CI: 0.21, 0.40<br>Median: 0.21<br>Range: 0.003-0.99	0.4
**Survival Time**	Mean: 158.5±49.1<br>95% CI: 51.6, 265.5<br>Median: 78<br>Range: 22-669	Mean: 171.2±25.6<br>95% CI: 119, 223.3<br>Median: 121.5<br>Range: 47-767	0.8
**Time to Progression**	Mean: 103.3±26.1<br>95% CI: 43.1, 163.6<br>Median: 65<br>Range: 34-238	Mean: 89.1±14.2<br>95% CI: 60.1, 118.2<br>Median: 60.5<br>Range: 29-321	0.6

2 patients in Cohort 1, 3 patients in Cohort 4, one patient in Cohort 5, 3 patients in Cohort 6A, 2 patients in Cohort 7, 1 patient in Cohort 8, 1 patient with intent to receive APVO436 according to an alternative exploratory schedule who developed a Grade 1 neurotoxicity with transient confusion after 1.5 mcg total dose (SAE due to hospitalization for diagnostic workup and observation), and none of the patients in the highest dose Cohorts 9 or 10 developed SAE.

Table S10. APVO436-related neurotoxicity occurring in patients treated with APVO436 in Phase 1B Study 5001

Patient No.	Cohort#	AE Reported Term (CTCAE Grade)	Concomitant CRS (Yes/No)	Start Date (CxDx); number of days from IC	Total Duration (Days)	Relatedness with APVO436	AE Outcome	Action Taken with APVO436
UPN12	4	Headache (1)	Yes	C1D22; 33	2	Related	Resolved	None
		Tremor (1)		C1D2; 12	1	Related	Resolved	None
		Confusion (1)		C2D1; 46	>12	Related	Not Resolved	DPD
UPN20	6A	Dizziness (1)	Yes	C8D22; 241	6	Related	Resolved	None
		Lethargy (1)		C8D15; 234	13	Related	Resolved	None
UPN28	6B	Insomnia (1)	No	C1D11; 18	NA	Related	Not Resolved	None
UPN31	7	Confusion (3)	Yes	C1D1; 120	1	Related	Resolved	None
UPN46	Other	Neurotoxicity (1); Confusion (1); Memory loss (1)	No	C1D1; 10	2	Related	Resolved	DPD

AE: Adverse event; CRS: cytokine release syndrome; ICF: Informed consent; DPD: drug permanently discontinued; NA: not applicable; C: cycle; D: Day

Table S11. Predictors of APVO436-related Neurotoxicity and Its Lack of Impact on Overall Survival

	Neurotoxicity<br>N=5	No Neurotoxicity<br>N=41	P-Value
**Age (Years)**	Mean: 71.4 ±3.0<br>95% CI: 63.0, 79.8<br>Median: 73<br>Range: 61-78	Mean: 64.68±2.14<br>95% CI: 60.37, 69.00<br>Median: 67<br>Range: 18.00-82.00	0.3
**Gender**	M: 40 %<br>F: 60%	M: 53.7%<br>F: 46.3%	0.6
**Race**	C: 80%<br>HL: 0%<br>W-HL: 20%<br>A: 0%<br>B: 0%	C: 70.7%<br>HL: 2.4%<br>W-HL: 14.6%<br>A: 4.9%<br>B: 7.3%	0.8
**Dose (µg/kg)**	Mean: 0.2±0.1<br>95% CI: 0.02146, 0.5<br>Median: 0.1490<br>Range: 0.05-0.4	Mean: 0.3±0.0<br>95% CI: 0.2, 0.4<br>Median: 0.2<br>Range: 0.003-1.0	0.7
**Survival Time (Days)**	Mean: 190.8±6<br>95% CI: 21.5, 360.1<br>Median: 246<br>Range: 34-316	Mean: 164.6±24.7<br>95% CI: 114.7, 214.4<br>Median: 109<br>Range: 22-767	0.7
**ALC x10^3/µL**	Mean: 1.0±0.4<br>95% CI: 0.02, 2.04<br>Median: 0.61<br>Range: 0.21-2.0	Mean: 0.9±0.1<br>95% CI: 0.6, 1.19<br>Median: 0.7<br>Range: 0.01-4.4	0.8
**% Lymphocytes**	Mean: 25.3±5.9<br>95% CI: 8.9, 41.6<br>Median: 32.4<br>Range: 5-36	Mean: 31.7±3.6<br>95% CI: 24.5, 38.9<br>Median: 25<br>Range: 2-78	0.5

BMI: body mass index; M: male; F: female; C: Caucasian; HL: Hispanic or Latino; A: Asian; B: Black or African-American; µg: microgram; ALC: absolute lymphocyte count

Table S12 Predictors of Favorable Response and Its Impact on Time to Progression of Leukemia

	Favorable Response N=8	No Favorable Response N=31	P-Value
**Age (years)**	Mean: 71.3 ±3.9<br>95% CI: 62.1, 80.4<br>Median: 74.5<br>Range: 47-82	Mean: 61.8±2.5<br>95% CI: 56.7, 66.8<br>Median: 65.0<br>Range: 18.00-81.00	0.079
**Gender**	M: 75%<br>F: 25%	M: 42%<br>F: 58%	0.095
**Race**	C: 75%<br>HL: 0%<br>W-HL: 0%<br>A: 12.5%<br>B: 12.5%	C: 67.7%<br>HL: 6.5%<br>W-HL: 19.4%<br>A: 0%<br>B: 6.4%	0.4
**Cumulative Dose (µg)**	Mean: 418.5±91.3<br>95% CI: 202.7, 634.3<br>Median: 442.5<br>Range: 9.1-816.0	Mean: 79.5±19.3<br>95% CI: 40.1, 118.9<br>Median: 43.5<br>Range: 1.2-495.0	<0.0001
**Cumulative Dose (µg/kg)**	Mean: 6.1±1.5<br>95% CI: 2.5, 9.6<br>Median: 5.4<br>Range: 0.1-11.8	Mean: 1.1±0.2<br>95% CI: 0.6, 1.6<br>Median: 0.7<br>Range: 0.01-5.69	<0.0001
**Highest Dose (µg/kg)**	Mean: 0.3±0.1<br>95% CI: 0.1, 0.6<br>Median: 0.3<br>Range: 0.004-0.721	Mean: 0.241±0.049<br>95% CI: 0.1, 0.3<br>Median: 0.1<br>Range: 0.003-0.990	0.3
**Survival Time (Days)**	Mean: 246.0±37.82<br>95% CI: 156.6, 335.4<br>Median: 238.0<br>Range: 110.0-395.0	Mean: 156.5±31.22<br>95% CI: 92.8, 220.3<br>Median: 89.0<br>Range: 22.0-767.0	0.2
**Time to Progression (Days)**	Mean: 182.4±25.7<br>95% CI: 119.5, 245.4<br>Median: 169<br>Range: 103-288	Mean: 52.0±3.3<br>95% CI: 45.2, 58.9<br>Median: 46<br>Range: 29-92	<0.0001

Table S13: Patient Characteristics, Demographic Features, and Treatment Outcome for APVO436-Treated MDS Patients

						BM Involvement					Treatment Outcome				
**Patient ID**	**Cohort**	**# of APVO 436 Doses**	**Diagnosis**	**Age/Sex/Race**	**Previous Therapies (Number: List)**	**Cellularity**	**Percent Myeloblasts-PRE**	**Percent Myeloblasts-POST**	**Karyotype/Mutations**	**Best Overall Response**	**Time to Progression (days)**	**C1D1 to EOT**	**Time to Death or Hospice**	**Survival Status at last FU**	**Cause of death**
UPN09	3	4	MDS	74/M/C	2: Decitabine; Exp x1	20	1	NE	del5q,add8q22, dup9q13q33, add11q24	NE	NA	34	47	D	Sepsis(NR)
UPN13	4	43	MDS	77/F/C	1: Decitabine	95	4	4(C10D22)	Unknown/ ASXL1, STAG2	SD	321	308	330	D	PD(NR)
UPN23	6A	18	MDS	80/M/C	1: AZA	10	7.5	2.4(C2D1)	Unknown	Marrow CR - SD	138	129	138	D	PD, IFI(NR)
UPN32	7	13	MDS	75/M/C	3:GCLAM; Vyxeos; Decitabine	20-30	11.3	0(C2D1)	11q-,-7/None	Marrow CR -SD	104	62	138	D	PD(NR)
UPN34	8	26	MDS	69/M/A	2: Venetoclax; AZA	ND	5	5(C2D1)	del(12p)/ND	SD	211	198	232	A	NA
UPN39	9	21	MDS	**75**/M/C	1:AZA	15	8.2	2(C2D1)	46,XY/ND	Marrow CR -SD	>147	>167	>167	A	NA
UPN45	10	6	MDS	74/F/C	1: AZA	20	6	6(C3D1)	t(1;2)/ND	SD	>106	NA	>106	A	NA

C2D1: Cycle 2 Day 1; C10D22: Cycle 10, Day 22; NE: Not evaluable; AZA: Azacitidine; GCLAM: the combined use of granulocyte colony-stimulating factor (G-CSF), cladribine, high-dose cytarabine, and mitoxantrone; Vyxeos (CPX-351), a liposomal formulation of cytarabine and daunorubicin at a fixed 5:1 molar ratio; MDS: Myelodysplastic Syndromes; PD: Progressive disease; ;SD: stable disease; IFI: invasive fungal infection; NR: not related to APVO436; NA: not applicable; ND: not determined; M: male; F: female; C: Caucasian; A: Asian; Exp. Experimental drug

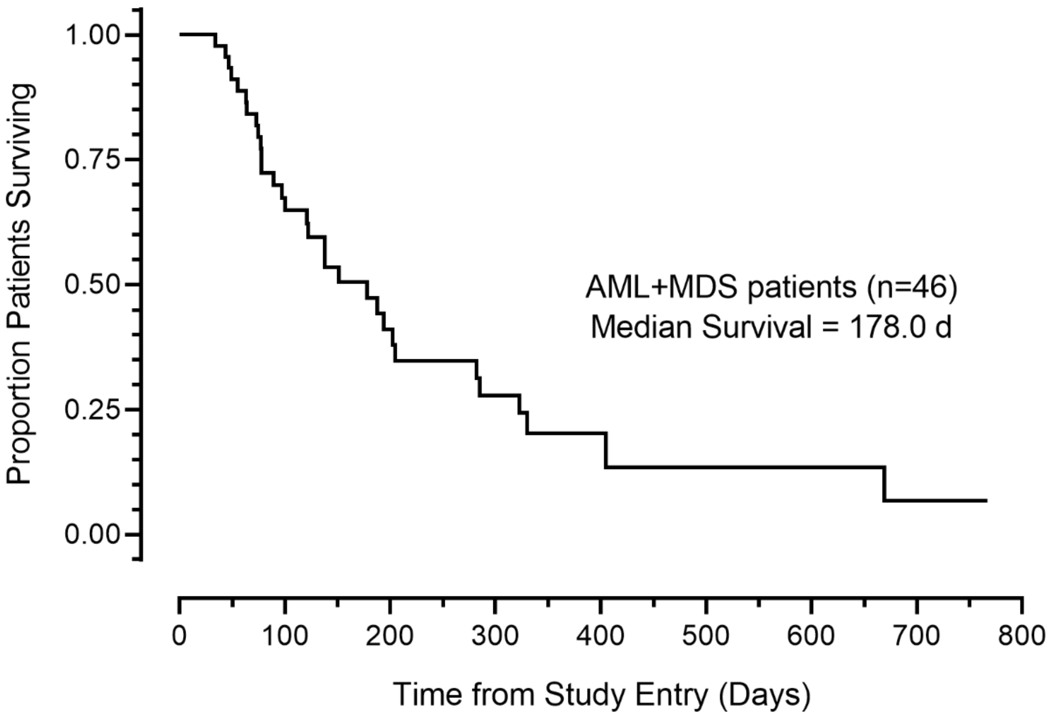

Figure S1. Survival Outcome of AML/MDS Patients. Depicted are the overall survival curves of the 46 R/R AML/MDS patients treated with APVO436.
